# 3D-MSNet: a point cloud-based deep learning model for untargeted feature detection and quantification in profile LC-HRMS data

**DOI:** 10.1093/bioinformatics/btad195

**Published:** 2023-04-18

**Authors:** Ruimin Wang, Miaoshan Lu, Shaowei An, Jinyin Wang, Changbin Yu

**Affiliations:** Fudan University, Shanghai 200433, China; School of Engineering, Westlake University, Hangzhou, Zhejiang Province 310030, China; Shandong First Medical University & Shandong Academy of Medical Sciences, Jinan, Shandong Province 250021, China; School of Engineering, Westlake University, Hangzhou, Zhejiang Province 310030, China; Shandong First Medical University & Shandong Academy of Medical Sciences, Jinan, Shandong Province 250021, China; Zhejiang University, Hangzhou, Zhejiang Province 310058, China; Fudan University, Shanghai 200433, China; Shandong First Medical University & Shandong Academy of Medical Sciences, Jinan, Shandong Province 250021, China; School of Life Sciences, Westlake University, Hangzhou, Zhejiang Province 310030, China; Shandong First Medical University & Shandong Academy of Medical Sciences, Jinan, Shandong Province 250021, China; Zhejiang University, Hangzhou, Zhejiang Province 310058, China; School of Life Sciences, Westlake University, Hangzhou, Zhejiang Province 310030, China; Shandong First Medical University & Shandong Academy of Medical Sciences, Jinan, Shandong Province 250021, China

## Abstract

**Motivation:**

Liquid chromatography coupled with high-resolution mass spectrometry is widely used in composition profiling in untargeted metabolomics research. While retaining complete sample information, mass spectrometry (MS) data naturally have the characteristics of high dimensionality, high complexity, and huge data volume. In mainstream quantification methods, none of the existing methods can perform direct 3D analysis on lossless profile MS signals. All software simplify calculations by dimensionality reduction or lossy grid transformation, ignoring the full 3D signal distribution of MS data and resulting in inaccurate feature detection and quantification.

**Results:**

On the basis that the neural network is effective for high-dimensional data analysis and can discover implicit features from large amounts of complex data, in this work, we propose 3D-MSNet, a novel deep learning-based model for untargeted feature extraction. 3D-MSNet performs direct feature detection on 3D MS point clouds as an instance segmentation task. After training on a self-annotated 3D feature dataset, we compared our model with nine popular software (MS-DIAL, MZmine 2, XCMS Online, MarkerView, Compound Discoverer, MaxQuant, Dinosaur, DeepIso, PointIso) on two metabolomics and one proteomics public benchmark datasets. Our 3D-MSNet model outperformed other software with significant improvement in feature detection and quantification accuracy on all evaluation datasets. Furthermore, 3D-MSNet has high feature extraction robustness and can be widely applied to profile MS data acquired with various high-resolution mass spectrometers with various resolutions.

**Availability and implementation:**

3D-MSNet is an open-source model and is freely available at https://github.com/CSi-Studio/3D-MSNet under a permissive license. Benchmark datasets, training dataset, evaluation methods, and results are available at https://doi.org/10.5281/zenodo.6582912.

## 1 Introduction

Untargeted liquid chromatography–mass spectrometry (LC-MS) is widely used in composition profiling in metabolomics and proteomics (Fiehn 2002; Aebersold and Mann 2003; Wishart 2016). In full-scan and data-dependent acquisitions (DDA), analytes are fully acquired as time-series spectra with three dimensions, retention time (RT), mass-to-charge (*m*/*z*), and intensity (*I*). Thousands of analytes are eluted at different times and acquired as 3D Gaussian-like features, which are different in volumes and shapes, mixed with noise, and overlap with each other. Due to the high-dimension, high-complexity, and high-throughput nature of mass spectrometry (MS) data, accurate feature extraction is a key challenge in untargeted data interpretation (Tautenhahn et al. 2008).

Many untargeted analytical tools and software packages were developed to extract features accurately from the complex raw data. These methods can be primarily divided into three categories: 2D analysis, 2.5D analysis, and 3D analysis. Most methods analyze MS data dimension by dimension in 2D and 2.5D to reduce the computation time and complexity. 2D methods, such as MS-DIAL (Tsugawa et al. 2015), MZmine 2 (Pluskal et al. 2010), XCMS Online (Tautenhahn et al. 2012), and PeakOnly (Melnikov et al. 2020), first perform 2D mass detection on spectra, then extract the extracted ion chromatograms according to the mass detection result, and finally perform 2D peak detection on EICs. 2.5D methods, such as MaxQuant (Cox and Mann 2008) and Dinosaur (Teleman et al. 2016), first perform mass detection on spectra and then assemble centroids in adjacent scans into 3D hills. In these methods, the data distribution of *m*/*z* dimension is not directly involved in feature discovery. Although the dimensionality reduction in the *m*/*z* dimension brings less computational complexity and faster computation speed, the incomplete analysis of the data distribution affects the accuracy of the feature detection results.

In the last decades, more and more methods attempted to perform 3D analysis and evaluate the *m*/*z* and RT information simultaneously for better feature detection and quantification. Since the signal points of MS data are sparsely distributed in 3D space with high throughput and complexity, direct analysis of raw MS data is challenging. Woldegebriel and Vivó-Truyols (2015) and Woldegebriel and Derks (2017) perform feature detection by estimating the peak probability for each gridded point with the Bayesian probabilistic model. They calculated the posterior probability based on the assumption that features obey the 2D Gaussian distribution. DeepIso (Zohora et al. 2019) and SeA-M2Net (Zhao et al. 2021) consider the gridded data as images and perform feature detection as an object detection task. Bounding boxes of isotope patterns are detected by convolutional neural networks, on which features are subsequently roughly quantified. PointIso (Zohora et al. 2021) considers gridded data as point clouds and applied a deep neural network to predict whether each gridded signal point belongs to a feature and to which feature it belongs. However, all existing 3D analysis methods suffer from insufficient feature detection and quantification accuracy. To facilitate analysis, existing 3D methods reduce data volume and complexity before feature extraction by gridding the raw MS data into a uniformly distributed form. The data precision decreases significantly in the equidistant grid transformation. Although the data precision can be theoretically maintained with smaller grid widths, the amount of the transformed interpolated data is much more considerable than the original and requires unaffordable computational space and time. In addition, these methods mainly focus on better feature detection, ignoring the need for accurate feature quantification. After feature probability and region proposal, there is still a demand for effective feature quantification methods to determine integration boundaries and separate overlapping features.

**Figure 1.**
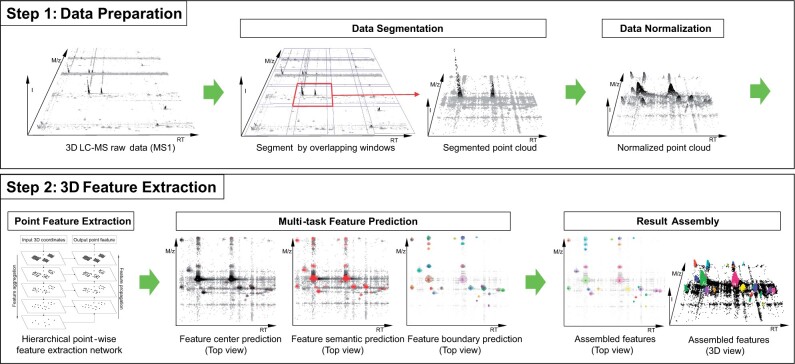
The workflow of 3D-MSNet. 3D-MSNet analyzes data in two main steps: data preparation and 3D feature extraction. In the data preparation step, LC-MS raw data are segmented into point clouds and then the point clouds are normalized to reduce variances in feature shape and volume. In the 3D feature extraction step, firstly, point clouds are analyzed by a hierarchical deep neural network to extract local spatial point features. Then, the extracted point features are analyzed by multitask neural networks for feature center prediction, feature semantic prediction, and feature boundary prediction. Finally, we use a result assembly mechanism to assemble the prediction results to final features and map the features to raw LC-MS data.

To achieve precise MS data quantification and avoid information loss in data transformation, in this article, we propose a novel method to fully utilize the 3D distribution of MS data, named as 3D-MSNet. 3D-MSNet is a single-stage multi-task deep learning-based model for point-wise 3D feature detection and quantification on profile full-scan and DDA data (Fig. 1). Instead of analyzing centroided or gridded data, 3D-MSNet enables direct spatial analysis of lossless 3D MS data for the first time. The inspiration for 3D-MSNet comes from the native point-cloud-like MS data structure. A point cloud is composed of a set of data points in space and is one of the primary representations of 3D objects and scenes. In the field of computer vision, deep neural networks have proven themselves effective in point cloud analysis for scenarios such as autonomous driving (Hu et al. 2019) and 3D scene understanding (Yang et al. 2019). 3D-MSNet considers features as 3D objects and performs feature detection and quantification as a point cloud instance segmentation task. In comparison with popular metabolomics and proteomics untargeted software, 3D-MSNet achieved significant improvements in feature detection and quantification with higher accuracy and stability on all benchmark datasets. Furthermore, 3D-MSNet has high feature detection robustness and can be widely applied to profile MS data acquired with various high-resolution mass spectrometers with various resolutions.

## 2 Materials and methods

### 2.1 Evaluation datasets

**Metabolomics datasets.** We used the two datasets published in Li et al. (2018), the TripleTOF 6600 dataset and the QE HF dataset. These two datasets were specifically created for the comprehensive evaluation of untargeted metabolomics software in terms of feature detection, quantification, and discriminating marker selection. The TripleTOF 6600 and QE HF datasets were acquired with full-scan methods on different HRMS platforms AB SCIEX TripleTOF 6600 interfaced with Shimazu L30A UPLC and Thermo Q Exactive HF with Dionex UltiMate 3000 HPLC, respectively. A pair of standard mixtures (SA, SB) were prepared for both datasets, composed of seven differential groups of compounds (Gd1, Gd2, Gd3, Gm, Gd4, Gd5, Gd6) prepared at different relative concentration ratios of (1/16, 1/4, 1/2, 1/1, 2/1, 4/1, 16/1) in SB:SA. Each of the mixtures had four replicates in the TripleTOF 6600 dataset and five replicates in the QE HF dataset. Benchmark libraries were provided along with the datasets and contained 970 and 836 features, manually selected and integrated from targeted analysis results of the TripleTOF 6600 dataset and the QE HF dataset, respectively.

**Proteomics dataset.** We used the Orbitrap XL dataset (ProteomeXchange, dataset PXD001091) (Chawade et al. 2015) for 3D-MSNet evaluation. This DDA dataset was acquired on a nanoflow liquid chromatography coupled to a Thermo LTQ Orbitrap XL ETD mass spectrometer. Synthetic potato and human peptides were spiked into stable and non-variable *Streptococcus pyogenes* strain SF370 background at 12 dilution levels. Four or seven replicates were acquired at each dilution level for a total of 57 injections. For each injection, 2 µl of the sample was injected at a constant flow of 300 nl/min with a gradient of 97% of Solvent A from 0 to 5 min, 65% A at 95 min, and 10% A from 98 to 108 min.

### 2.2 Training dataset

As far as we know, no public dataset provides 3D point-wise feature annotation results. We manually annotated a 3D feature instance dataset to train and evaluate our model, named the 3DMS dataset.

**3DMS dataset.** The 3DMS dataset contains 306 point clouds with 1 007 297 annotated signal points and 3727 annotated features. The point clouds were extracted from the first replicate of mixture SA (SampleA_1) in the metabolomics TripleTOF 6600 dataset, with fixed extraction windows at 0.8 Da *m*/*z* width and 6 min RT width. For clearer labeling, signals with intensity lower than 128 were considered as noise and not inherited to the extracted point clouds. All point clouds were point-wise labeled using the annotation software from BasicAI (www.basic.ai). Each signal point was assigned an instance label indicating whether the point belonged to a feature, and to which feature it belonged. All annotation results were checked by three experts and can be freely downloaded as .csv files at Zenodo (https://doi.org/10.5281/zenodo.6582912).

### 2.3 Input preparation

3D-MSNet supports two raw file formats: mzML and Aird (Lu et al. 2022). We used MSConvert 3.0.21288 (Chambers et al. 2012) to convert vendor MS files into mzML format and used Pyteomics (Levitsky et al. 2019) to read the converted files.

3D-MSNet segments MS data into point cloud blocks for processing (Supplementary Fig. S1). Although the network is designed with no limitation on the input point number, the entire input of the MS point cloud is unaffordable due to the limitation in computation resources. 3D-MSNet calculates segmentation windows in two steps. Firstly, calculate initial windows according to the manually set segmentation width. The size of the segmentation window can be adjusted according to the capacity of GPU video memory. To avoid feature detection errors on segmentation boundaries, in the second step, 3D-MSNet expands initial windows in both dimensions according to the expansion widths set by the user. The expansion width in each dimension should be set larger than the largest feature width in the corresponding dimension. In this way, features centered within the initial window are fully contained in the extended window. Optionally, noise signal points can be filtered in this process by setting a fixed intensity threshold to reduce subsequent analysis time. In the final merging of quantitative results, for each point cloud, only the detected features whose centers were within the initial window are retained, which ensures the comprehensiveness and nonrepetition of the feature extraction results.

Normalization was performed for better model generalization. On the intensity dimension, base two logarithmic transformation is applied to point intensities, while obvious feature shapes are retained in different abundances. On the RT dimension, data are centered to zero and normalized according to the typical RT full width at half maximum (FWHM). On the *m*/*z* dimension, data are also centered to zero and normalized by the theoretical *m*/*z* FWHM. The theoretical *m*/*z* FWHM is calculated from three parameters: mass analyzer principle, an *m*/*z* value, and the resolution at the *m*/*z*. 3D-MSNet supports time-of-flight (TOF) and Orbitrap mass analyzers, which are different in theoretical resolution calculation. All normalization operations are reversible so that the accuracy is maintained.

### 2.4 Network structure

**Figure 2.**
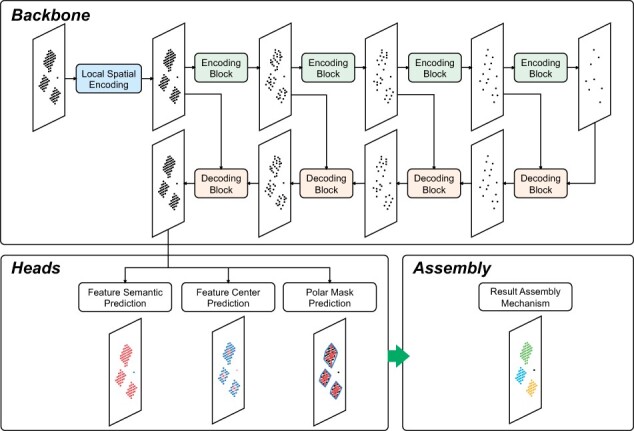
The network structure of 3D-MSNet.

3D-MSNet improves the accuracy of feature detection and quantification by means of direct and complete utilization of raw MS data. The architecture of 3D-MSNet is illustrated in Fig. 2. 3D-MSNet is a multitask end-to-end deep neural network composed of a feature extraction backbone, three prediction branches, and a prediction result assembly mechanism.

The backbone of 3D-MSNet is well-designed for point-wise hierarchical feature extraction. After data partition and normalization, 3D MS point clouds are fed into the network backbone. To extract hierarchical spatial features, first, the Local Spatial Encoding Block is applied to extract the local spatial features of each point. The Local Spatial Encoding Block uses a shared multilayer perception (MLP) to encode nearest neighbor 3D coordinates to spatial features. Then, multiple Encoding Blocks are applied for hierarchical feature encoding. In each Encoding Block, point features are aggregated by intensity probability sampling and point-wise interpolated convolution. While point clouds are sampled into fewer points, each sampled point obtains higher-level features by aggregating features from a larger receptive field. Next, multiple Decoding Blocks are applied for propagating the extracted hierarchical features to raw MS points. In each Decoding Block, point features abstracted from different levels are combined by nearest-neighbor interpolation and concatenation, and then a shared MLP is applied to transform concatenated features to final decoded features. The detailed implementation methods of Local Spatial Block, Encoding Block, and Decoding Block are illustrated in Supplementary Fig. S2.

**Figure 3.**
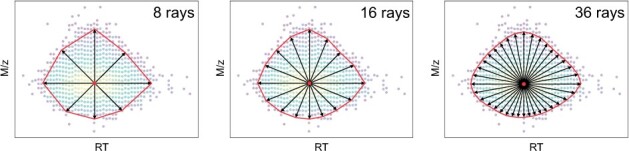
Polar mask representation at different ray numbers. By increasing the ray number from 8 to 36, the polar mask has higher boundary description accuracy. 3D-MSNet uses 36-ray polar masks to represent feature boundaries.

3D-MSNet has three prediction branches: feature semantic prediction, feature center prediction, and polar mask prediction. All prediction branches are implemented with fully connected networks. In the feature semantic prediction branch, the point-wise probability of belonging to a feature is predicted to distinguish features and noises. In the feature center prediction branch, the point-wise probability of being the center of a feature is predicted to distinguish different features. In the polar mask prediction branch, the point-wise feature contour is predicted to demarcate the feature quantification area. 3D-MSNet does not directly predict the instance label of each point, because the instance label of each point is determined by the probability of belonging to all instances and a fixed screening threshold, which leads to confusing spatially discontinuous instance segmentation results. Since features are compactly distributed, 3D-MSNet predicts a polar mask for each feature to obtain more robust quantification results, which is a 2D feature contour on the *m*/*z* and RT plane represented by a center point and multiple equiangular rays in Polar Coordinates (Fig. 3). The feature intensity is the volume of the 3D peak within the predicted feature boundary. By predicting an explicit boundary for each feature, 3D-MSNet can separate overlapping features and obtains more robust quantification results. Complex feature extraction examples are shown in Supplementary Fig. S3 using the visualization method of Open3D (Zhou et al. 2018).

Finally, we designed an assembly mechanism to aggregate the predicted results of the above three branches. Potential center points are first selected by analyzing semantic and center prediction results. Then, the duplicate identified centers belonging to the same feature are screened out by analyzing the relationship between 3D coordinates and polar masks of potential center points. At last, intersected feature contours are separated among the polar masks of screened center points to avoid repeated quantification of each point. After the assembly of predicted results, all features in point clouds are accurately detected and quantified. For each detected feature, we use *m*/*z* and RT coordinates of the predicted center to represent the feature location and calculate the feature volume with points in the predicted polar mask.

### 2.5 Network training

3D-MSNet was built with Python (version 3.6.13), PyTorch (version 1.9), CUDA (version 11.1), and cuDNN (version 8.0.5) on Ubuntu (version 16.04) and further trained, validated, and tested on a computer with an Intel(R)_Core(TM)_i9-10900K CPU, 128GB memory, and a GeForce RTX 3090 GPU.

We used the self-annotated 3DMS dataset for network training and validation. The 3DMS dataset was randomly separated into the training set and validation set according to the ratio of 7:3. From the 306 annotated point clouds, 214 point clouds were selected for training and the rest 92 point clouds for validation.

To improve the model’s generalization ability, data augmentation was applied to the training dataset in the loading stage of every training epoch to increase the data amount and enrich the data diversity. In the training process of 3D-MSNet, data in all dimensions were scaled by a random factor of 0.5 to 2, and data in *m*/*z* and RT dimensions were added with random offsets. To improve the training efficiency and stability, in each training epoch, eight randomly selected annotated point clouds were fed to the model simultaneously as a batch. Low-intensity simulated chemical noises were filled alongside the real data for small point clouds to make the number of points consistent within each point cloud of the batch.

The loss functions of 3D-MSNet are summarized in Supplementary Table S1, and the accuracy functions are summarized in Supplementary Table S2. We used focal loss in the feature semantic prediction branch and the feature center prediction branch and used polar mask Intersection over Union (IoU) loss in the polar mask prediction branch. The total loss function is the weighted sum of all branch’s loss functions and optimized with the Adam optimizer by setting the initial learning rate at 0.0005 and weight decay at 0.01.

## 3 Results

### 3.1 Overfitting evaluation on 3DMS dataset

To evaluate the performance of 3D-MSNet training, we plotted the loss and accuracy curves of each prediction branch during the training process (Supplementary Fig. S4). As the training epoch increased, the loss of training and validation set decreased synchronously, and the accuracy increased synchronously, which proved that the training process was stable and there was no overfitting.

To further demonstrate that 3D-MSNet did not overfit during training, we evaluated the model pre-trained on the 3DMS dataset on three public benchmark datasets with different data distributions below. 3D-MSNet showed high accuracy on all datasets. The model learned general feature point cloud distributions robustly, which proved that the training was not overfitting.

### 3.2 Performance evaluation on metabolomics datasets

We compared 3D-MSNet with five popular untargeted metabolomics software: MarkerView (version 1.3.1), Compound Discoverer (version 3.2.0.421), MS-DIAL (version 4.70), MZmine 2 (version 2.53), and XCMS Online (version 2.7.2), on both the TripleTOF 6600 dataset and the QE HF dataset in terms of untargeted feature detection, quantification, and discriminating marker selection. Software parameters were fine-tuned to maximize the identification rate and were summarized in Supplementary Tables S3 and S4. In the parameter setting, we used all software’s built-in alignment and gap-filling methods to achieve the best identification results and avoid the removal of isotopes and adducts to prevent the impact on the feature identification number. Due to the limitation of software functions, Compound Discoverer and MS-DIAL results are deisotoped, and MarkerView and 3D-MSNet results are not gap-filled. The first file (SampleA_1) was excluded from comparison to avoid the underlying improvements caused by manual annotation.

#### 3.2.1 Feature detection and quantification

In evaluation of feature detection and quantification performance, we used the feature libraries provided by the dataset, which contained 970 features in the TripleTOF 6600 dataset and 836 features in the QE HF dataset. Since the standard mixtures only differed in concentration, for each dataset, we first retained the consensus features detected in all samples. Then, the consensus features were matched to the library with *m*/*z* and RT tolerances at (0.01 Da, 0.5 min) on the TripleTOF dataset, and (0.005 Da, 0.3 min) on the QE HF dataset (Supplementary Fig. S5). To evaluate the quantification accuracy, we calculated the mean intensity of all replicates in each mixture and calculated the fold-change (SB:SA) of each matched feature. The features, whose fold-changes were within 20% tolerance of the theoretical and 2D measured range, were considered as accurately quantified features. The measured results were obtained by 2D targeted analysis and manual integration of library features using the MetaPro batch inspection tool (https://github.com/CSi-Studio/MetaPro).

**Figure 4.**
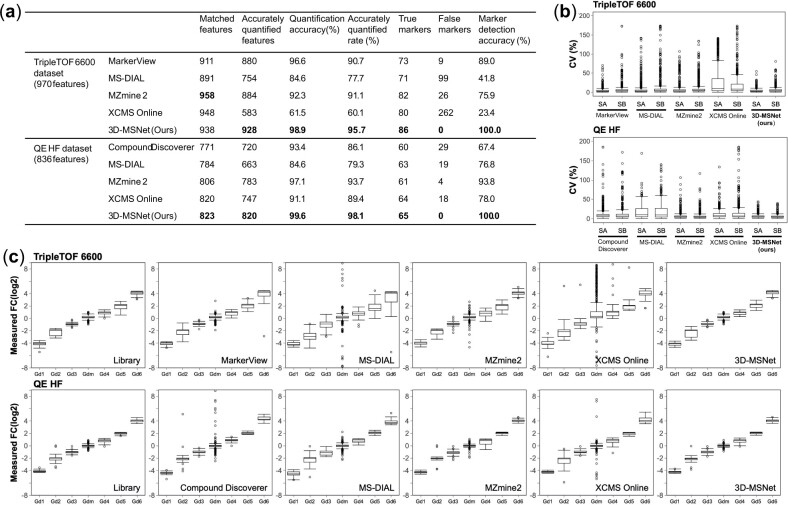
Evaluation results on the metabolomics TripleTOF 6600 and QE HF datasets. (a) Results table for feature detection, feature quantification, and marker detection. (b) Coefficient of variance (CV) distribution across technical replicates. (c) Relative concentration ratio between standard mixtures.

3D-MSNet achieved the best feature detection and quantification performance on both datasets (Fig. 4a). In the comparison results of the TripleTOF 6600 dataset, 3D-MSNet detected the highest number of accurately quantified features with a percentage of 95.7%, higher than MZmine 2 at 91.1% and MarkerView at 90.7%. As for quantification, 3D-MSNet achieved the highest quantification accuracy at 98.9%, higher than MarkerView at 96.6% and MZmine 2 at 92.3%. In comparison results of the QE HF dataset, 3D-MSNet had similar improvements on all statistical metrics and achieved substantially better performance than the other compared software. In terms of feature detection performance, 3D-MSNet detected the highest number of accurately quantified features with a percent at 98.1%, significantly higher than MZmine 2 at 93.7% and XCMS Online at 89.4%. In terms of feature quantification performance, 3D-MSNet achieved the highest quantification accuracy at 99.6%, higher than MZmine 2 at 97.1% and Compound Discoverer at 93.4%.

To evaluate the accuracy of quantification, we compared the measured relative quantification results with the theoretical results. The log fold-changes (SB:SA) of matched features between standard mixtures were divided into seven groups according to the theoretical concentration ratio (1/16, 1/4, 1/2, 1/1, 2/1, 4/1, 16/1) and plotted in Fig. 4c. 3D-MSNet achieved the highest relative quantification accuracy on both datasets and outperformed other popular software with the tightest relative quantification distribution and the least outlier. In comparison with the manually integrated 2D targeted quantification library result, 3D-MSNet reached the performance equivalent to manual annotation and showed distributions with high similarity, as the 2D extracted feature fractions were proportional to full 3D features under clean feature distributions. To evaluate the stability of quantification, we calculated the coefficient of variance (CV) values of quantification results of matched features among replicates (Fig. 4b). Compared with other popular software, our 3D-MSNet showed the highest stability with the lowest CV value on both datasets.

In evaluation of marker detection performance, we considered the consensus features whose fold-changes were out of 20% tolerance of the range of (0.5, 2) as markers (Supplementary Appendix S1). True markers are the markers whose theoretical fold-changes are out of the range of (0.5, 2), and the rest of the markers are identified as false markers. 3D-MSNet achieved the best marker detection performance. In the TripleTOF 6600 dataset, 3D-MSNet detected the highest number of true markers at 86 and detected 0 false markers with the highest marker detection accuracy rate at 100.0%, significantly higher than MarkerView at 89.0% and MZmine2 at 75.9%. In the QE HF dataset, 3D-MSNet detected the highest number of true markers at 65 and detected 0 false markers, achieving the highest marker detection accuracy rate at 100.0%, higher than MZmine 2 at 93.8% and XCMS Online at 78.0%. 3D-MSNet detected the highest number of true markers in both datasets without detecting any false positive markers, outperforming other alignment software with absolute leading accuracy.

### 3.3 Performance evaluation on proteomics dataset

We further evaluated 3D-MSNet on the Orbitrap XL dataset against four proteomics software: MaxQuant (version 2.0.3.1), Dinosaur (version 1.2.0), DeepIso, and PointIso. MaxQuant and Dinosaur are 2.5D traditional methods, and DeepIso and PointIso are 3D deep learning methods. We followed the parameter settings of MaxQuant and Dinosaur in the Orbitrap XL dataset paper and used default parameters and models for DeepIso and PointIso as recommended by their author. 3D-MSNet parameters are listed in Supplementary Table S5.

**Figure 5.**
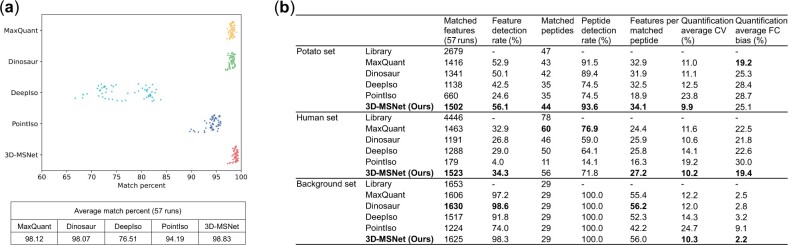
Evaluation results on the proteomics Orbitrap XL dataset. (a) Distribution of detection percent of high-confidence features of all files. Each point represents the match percentage of a file. (b) The feature extraction results of spike-in and background peptides.

First, we evaluated feature detection performance in each MS injection by matching untargeted feature detection result to high-confidence features. Following the approach of PointIso, MASCOT (version 2.5.1) identifications with peptide score >25 were considered as high-confidence features. *m*/*z* and RT tolerances used in matching were set to 0.01 Da and 0.4 min according to the match deviation distributions (Supplementary Fig. S6). Features whose *m*/*z* and RT ranges contained the high-confidence features were identified as successfully matched features (Supplementary Appendix S2). 3D-MSNet outperformed other methods and achieved the highest feature detection ratio (match percentage of high-confidence features) in most files (53 of 57) (Supplementary Tables S6–S8), and the highest feature detection rate in all the 12 samples with an overall average detection rate at 98.83%, higher than MaxQuant at 98.12% and Dinosaur at 98.07% (Fig. 5a). In the comparison of the correlation of quantitative results across different methods, 3D-MSNet showed high quantification accuracy in all files. The quantification correlations of 3D-MSNet with MaxQuant, Dinosaur, and DeepIso, were significantly higher than the correlations between any two of them (Supplementary Fig. S7).

Then, we used the spike-in and background peptide library to evaluate feature extraction performance under different dilution levels. The library was provided in the targeted selected reaction monitoring (SRM) dataset paired with the Orbitrap XL dataset, which shared the same LC platform and parameters. Since the Orbitrap XL dataset has significant RT and intensity variations in 57 injections, we introduced additional alignment and normalization processing. Limited by main text pages, the analysis process and results are detailed in Supplementary Appendix S3 and summarized and Fig. 5b. Considering the results of two spike-in sets (human set, potato set) and background set comprehensively, 3D-MSNet achieved the highest feature detection rate (match percentage of SRM library peptides) and quantitative stability (lowest average CV of each matched feature among technical replicates) with high quantitative accuracy (low average fold change (FC) bias among dilution levels).

For a more comprehensive evaluation not limited to selected features, we further evaluated the accuracy of the full feature detection results based on the distribution of the raw MS data. The distribution of untargeted feature detection results varied significantly among different software (Supplementary Fig. S8). We selected three regions with significantly different distributions and judged the correctness of the feature detection results by comparing with the original MS data. In the first region (Supplementary Fig. S9A), the long streaks along the RT dimension are chemical noises. 3D-MSNet showed the best performance in chemical noise discrimination, and all other compared methods had false positive detections. In the second region (Supplementary Fig. S9B), referring to the distribution of raw data, 3D-MSNet also achieved the best performance in short chemical noise discrimination. In the third region (Supplementary Fig. S9C), the long streak along the *m*/*z* dimension was caused by the rapid change of the proportion of liquid chromatography solvent. In experiment methods, the proportion of solvent A was rapidly reduced from 65% at 95 min to 10% at 98 min, and huge amounts of analytes were eluted into the mass spectrometer within a short period of time. The central dense region of the feature exhibited a chemical noise-like distribution along the *m*/*z* dimension. 3D-MSNet did not detect these anomalous signals as features and performed accurate noise-signal discrimination in all complex scenarios. Among the comparison methods, 3D-MSNet achieved the highest feature detection precision.

### 3.4 Generalization evaluation

The purpose of 3D-MSNet is to learn the feature distribution robustly and improve untargeted feature detection and quantification accuracy on profile LC coupled with high-resolution MS data. In all the evaluations, 3D-MSNet used the same model pre-trained on the 3DMS dataset. The TripleTOF 6600 dataset was acquired from the same sample set as the training 3DMS dataset by the same LC-MS platform. The QE HF dataset was acquired from the same sample set by a different LC-MS platform, which used a mass detector with different working principles and resulted in different feature shapes, feature volumes, and signal densities. The proteomics Orbitrap XL dataset was acquired from a different sample set and a different LC-MS platform, which resulted in completely different data distribution with the training 3DMS dataset. On all the three datasets, 3D-MSNet outperformed other software in terms of feature extraction and proved its effectiveness and robustness. We also evaluated proteomics software on the metabolomics datasets. The proteomics software detected fewer features than metabolomics software (Supplementary Table S10), which further highlighted the generalization ability of 3D-MSNet.

We further tested the generalization ability of 3D-MSNet on more datasets (Supplementary Fig. S10) with the same pre-trained model. These datasets were acquired by different mass spectrometers and acquisition methods (Li et al. 2018; Müller et al. 2020; Zhang et al. 2020), which resulted in different feature shapes, feature volumes, and signal densities. 3D-MSNet performed precise 3D feature extraction on all datasets, proving that 3D-MSNet learned the common characters of 3D features without overfitting. 3D-MSNet showed high robustness and versatility and can be applied to more MS data analysis scenarios that require 3D feature extraction.

### 3.5 Analysis time

We measured the running times of all comparison software on all evaluation datasets (Supplementary Table S9). Although 3D-MSNet uses huge amounts of spatial searching and encoding, it accelerates computations through code optimization and GPU parallel acceleration. 3D-MSNet took about 5 minutes per file when analyzing the TripleTOF 6600 dataset and about 20 min per file on the QE HF dataset and the Orbitrap XL dataset, as mass spectrometers with orbitrap analyzer acquiring analytes into more signal points. 3D-MSNet run significantly faster than other deep learning methods (about five times faster) and had a similar running time to traditional analysis methods. The computation times of MarkerView, Compound Discoverer, MS-DIAL, MZmine 2, and MaxQuant were measured on a Windows 10 computer with an Intel(R)_Core(TM)_i9-9880H CPU. The computation times of Dinosaur, DeepIso, PointIso, and 3D-MSNet were evaluated on an Ubuntu 16.04 computer with an Intel(R)_Core(TM)_i9-10900K CPU and a GeForce RTX 3090 GPU.

## 4 Conclusion

Feature extraction is fundamental to metabolomics LC-MS data interpretation. The accuracy of feature detection and quantification determines the rationality and credibility of subsequent statistical and biological analysis. In this study, we propose 3D-MSNet to learn the 3D feature distribution robustly and improve untargeted feature detection and quantification accuracy on profile LC-MS data. 3D-MSNet treats the feature extraction task on MS data as an instance segmentation problem on 3D MS point clouds. Using a multitask hierarchical deep neural network, 3D-MSNet enables direct spatial analysis of lossless MS data for the first time. Due to the comprehensive analysis of data distribution, 3D-MSNet showed obvious advantages in signal-noise discrimination and overlapping feature separation. In comparison with popular software, 3D-MSNet made consistent and significant improvements in feature detection and quantification accuracy on all the three metabolomics and proteomics benchmark datasets with competitive computational time.

3D-MSNet is a successful attempt to 3D analysis of MS data, providing a new way for more comprehensive MS data analysis. For now, 3D-MSNet only provides command-line-based interaction. In the future, we will develop a supporting graphical interface for 3D-MSNet to facilitate users to generate, check, and correct results.

## Supplementary Material

btad195_Supplementary_Data
