# Supplementary material for: 3D-MSNet: a point cloud-based deep learning model for untargeted feature detection and quantification in profile LC-HRMS data
Source: Bioinformatics. 2023 Apr 18;39(5):btad195. doi: 10.1093/bioinformatics/btad195 (PMC10181839; doi:10.1093/bioinformatics/btad195)
Supplement: btad195_Supplementary_Data [file btad195_supplementary_data.pdf]

Supplementary Information

3D-MSNet: A deep learning model for 3D feature instance segmentation on high-resolution mass spectrometry point clouds

Ruimin Wang, Miaoshan Lu, Shaowei An, Jinyin Wang and Changbin Yu

Figure S1. Determination of point cloud segmentation windows. (A) Raw LC-MS data. (B) Initial windows. (C) Expanded windows.

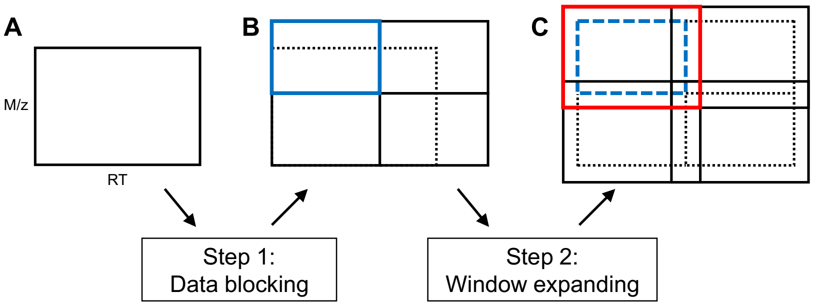

Figure S2. Detailed implementation of local spatial encoding block, encoding block, and decoding block in the 3D-MSNet backbone.

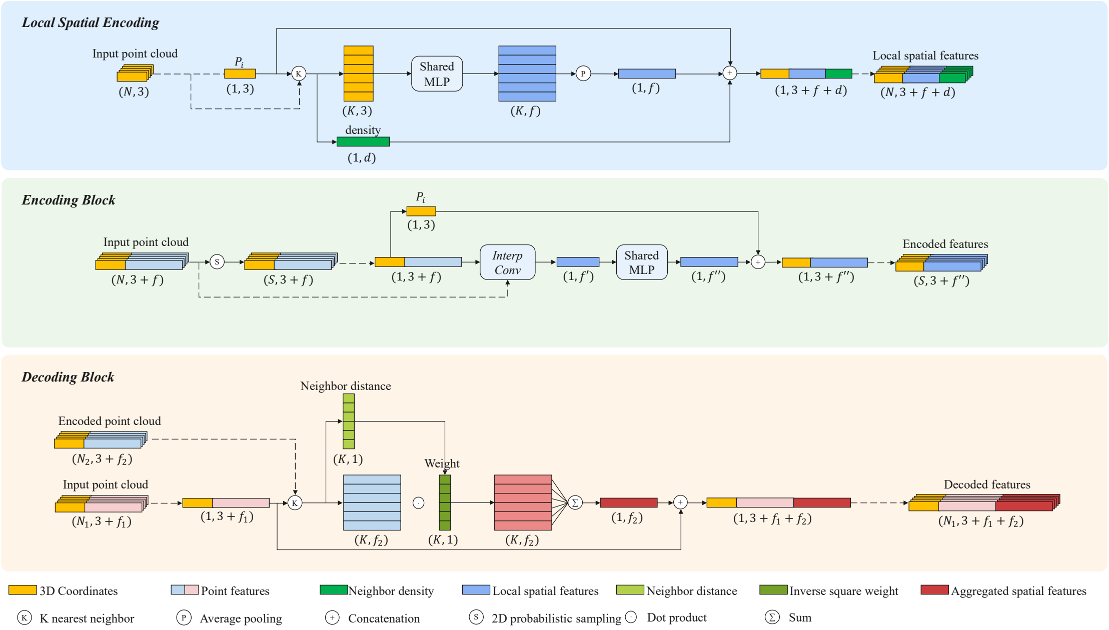

Figure S3. Feature extraction performance of 3D-MSNet on overlapping features.

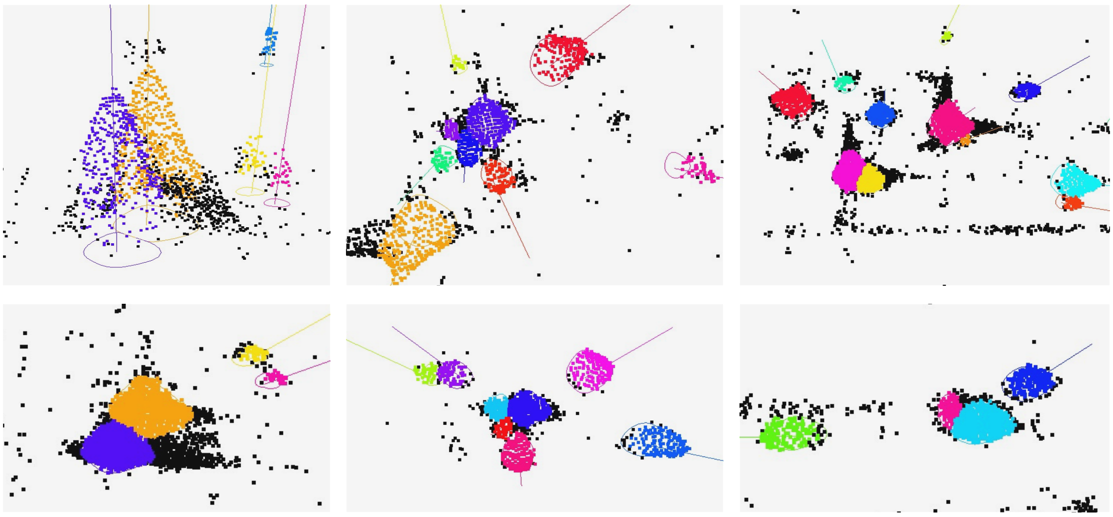

Figure S4. The loss and accuracy curves of 3D-MSNet in network training. (A) Loss curves in 3D-MSNet training. (B) Accuracy curves in 3D-MSNet training

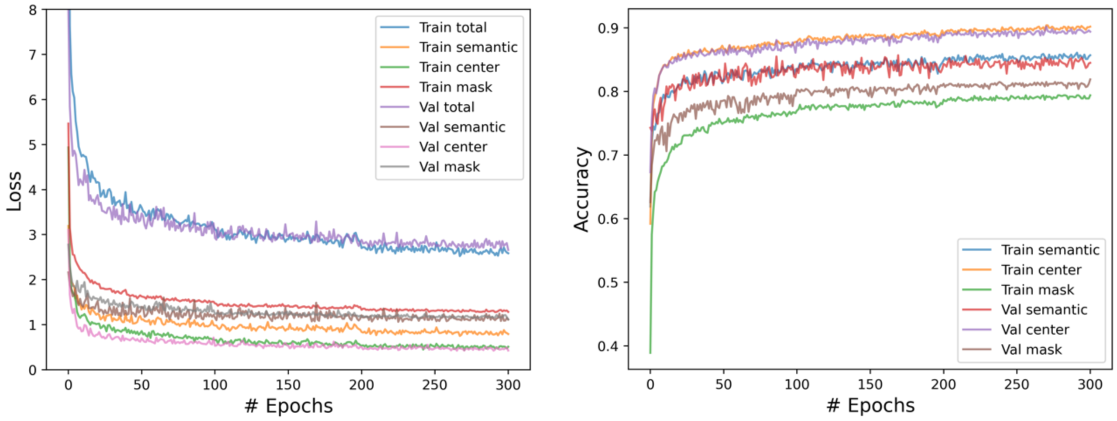

Figure S5. M/z and RT tolerance setting in evaluation of the metabolomics datasets.

Since the TripleTOF 6600 dataset and the QE HF dataset were acquired with different LC-MS platforms (the TripleTOF 6600 dataset was acquired by AB SCIEX TripleTOF 6600 interfaced with Shimazu L30A UPLC and the QE HF dataset was acquired by Thermo Q Exactive HF with Dionex UltiMate 3000 HPLC), the offsets for these two datasets were slightly different on both m/z and RT dimensions. In order to set a tight threshold and minimize additional false positive matchings, we plotted the offset distributions of the library compounds across different samples below. According to the offset distribution of matched target library compounds, we set RT and m/z tolerances at (0.5min, 0.01Da) for the TripleTOF 6600 dataset and (0.3min, 0.005Da) for the QE HF dataset to harvest high-precision comparison results.

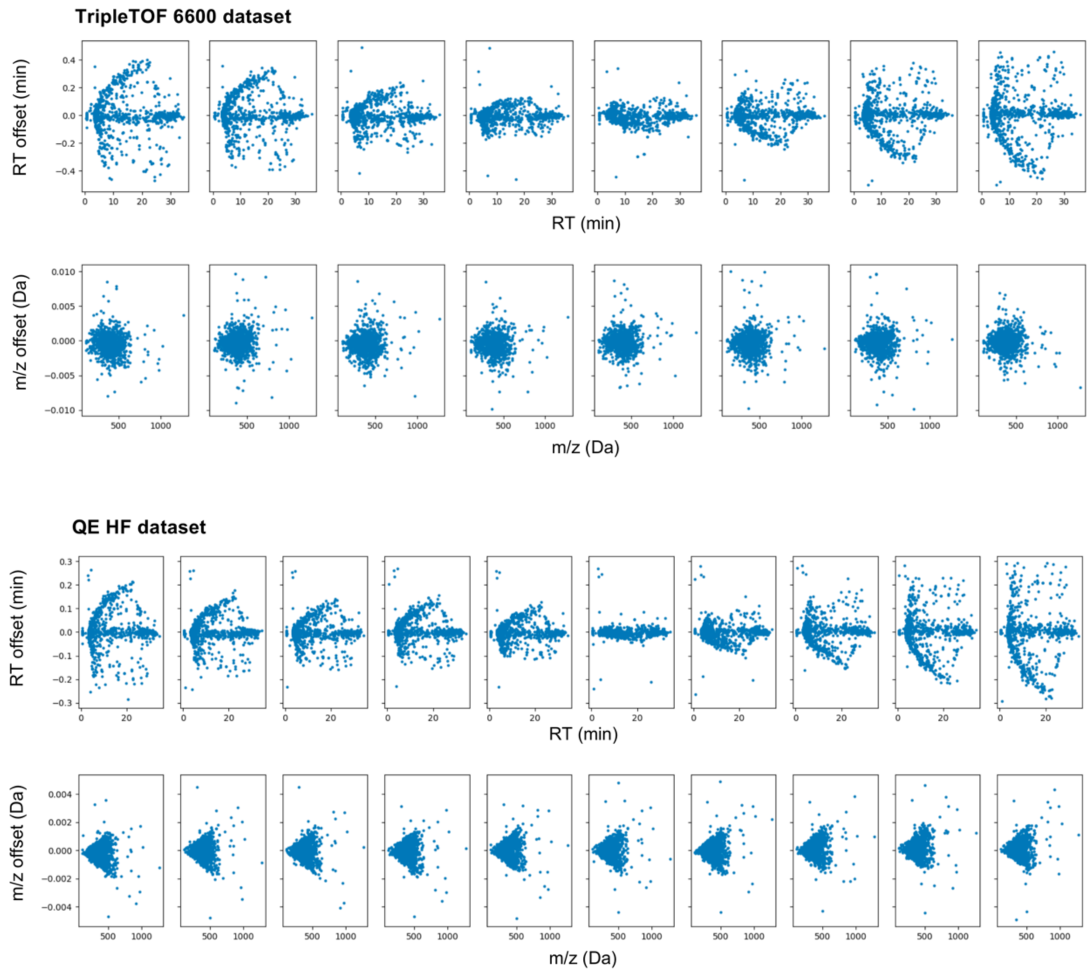

Figure S6. M/z and RT deviation distributions matched to MASCOT high-confidence features on the Orbitrap XL dataset. (A) RT deviation histogram for high-confidence matches. RT deviation = RT of the high-confidence MS2 spectrum – apex RT of the detected feature. (B) *M/z* deviation histogram for high-confidence matches. *M/z* deviation = precursor *m/z* of the high-confidence MS2 spectrum – *m/z* of the detected feature.

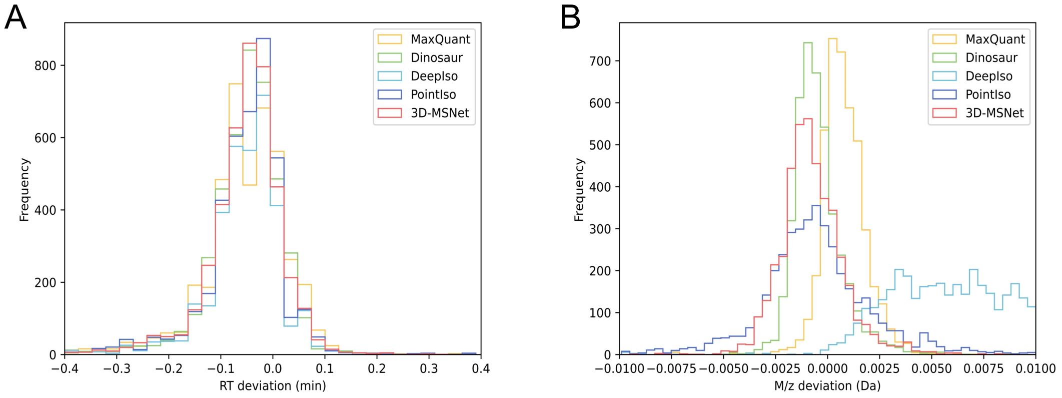

Figure S7. Distribution of quantitative correlations between software of all files.

Each point represents the quantitative Pearson correlation of a run.

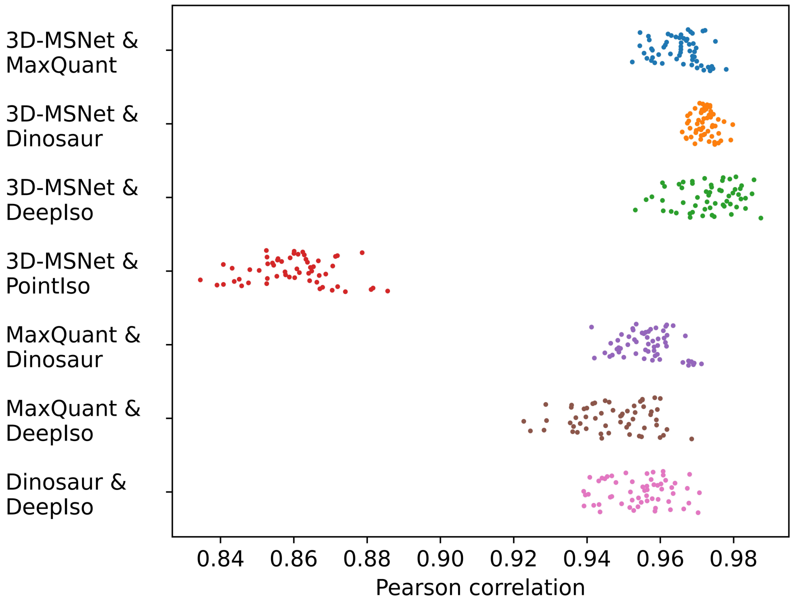

Average Pearson correlation (57 runs)						
3D-MSNet & MaxQuant	3D-MSNet & Dinosaur	3D-MSNet & DeepIso	3D-MSNet & PointIso	MaxQuant & Dinosaur	MaxQuant & DeepIso	Dinosaur & DeepIso
0.965	0.972	0.973	0.860	0.956	0.947	0.955

Figure S8. Feature detection map of the Orbitrap XL dataset. Untargeted feature detection results of the first file (130124_dilA_1_01) are plotted in figures below. MASCOT features are the high-confidence features with peptide score > 25. Each point in the figures represents a detected feature.

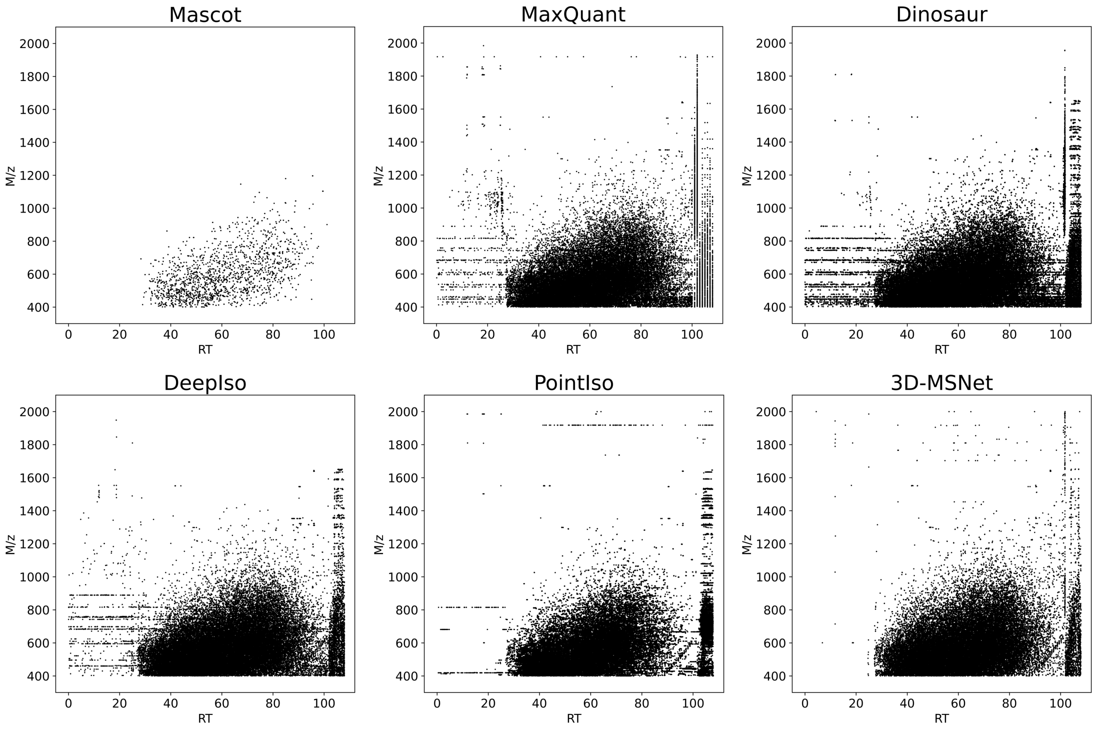

Figure S9. Zoom in view of raw data for regions with different feature detection distributions. We compared three regions where the distributions of 3D-MSNet feature detection results were different from other software.

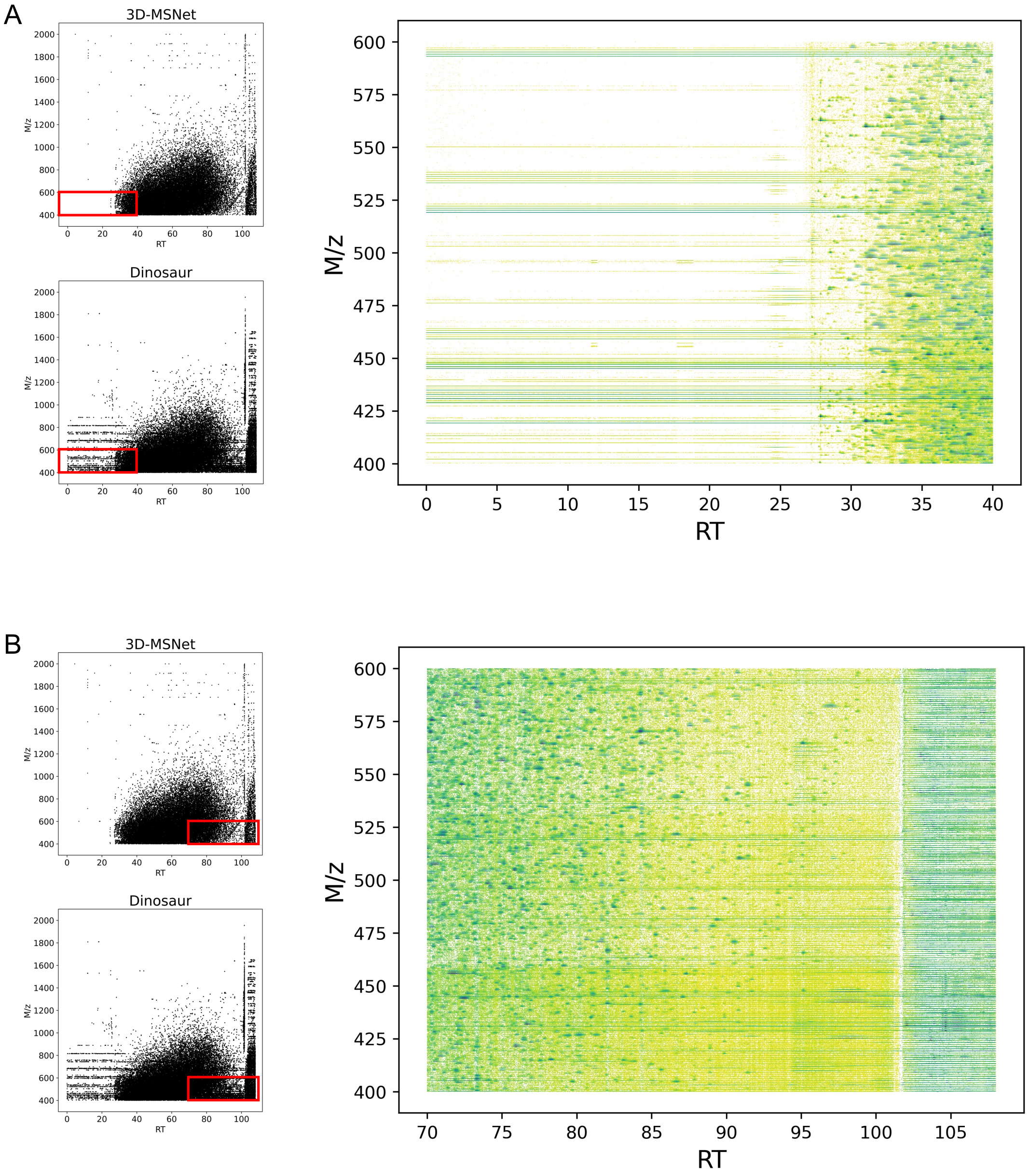

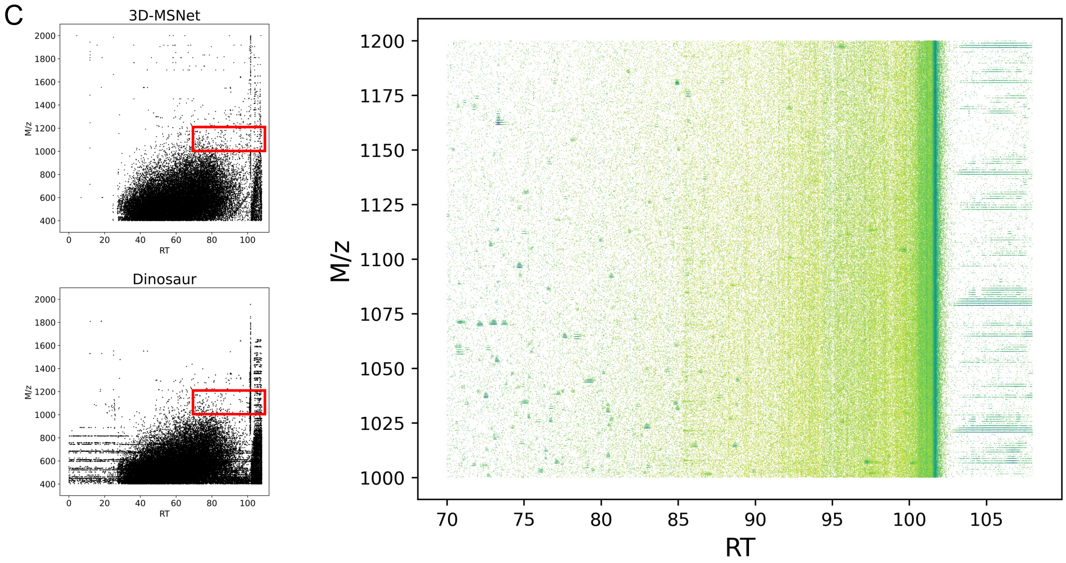
C
3D-MSNet
Dinosaur
M/z
RT
2000
1800
1600
1400
1200
1000
800
600
400
0
20
40
60
80
100
1200
1175
1150
1125
1100
1075
1050
1025
1000
70
75
80
85
90
95
100
105

Figure S10. Feature extraction performance of 3D-MSNet on profile data acquired by different mass spectrometers and acquisition methods. 35000@956Da means the mass spectrometry resolution is 35000 when the signal m/z is 956Da.

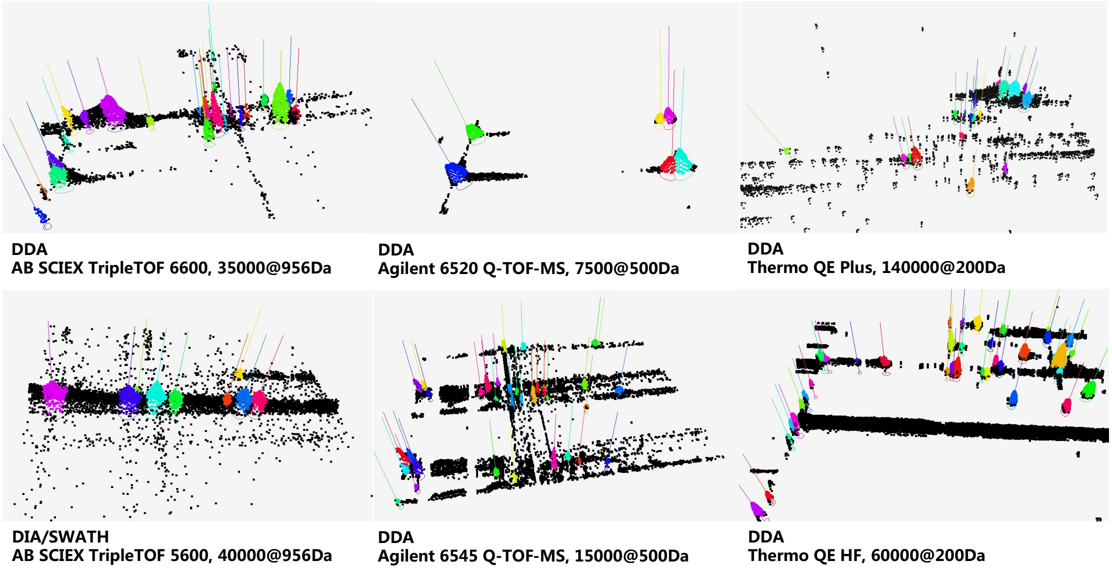

Table S1. The loss functions of 3D-MSNet. Semantic loss is the loss of the feature semantic prediction branch. Center loss is the loss of the feature center prediction branch. Polar mask loss is the loss of polar mask prediction branch. The total loss of 3D-MSNet is the weighted sum of the semantic loss, the center loss, and the polar mask loss.

Semantic loss (L_S)	$L_{S0} = Avg(-\hat{p}_i^2 \log(1-\hat{p}_i)), \quad i \in I_F$<br>$L_{S1} = Avg(-(1-\hat{p}_i)^2 \log \hat{p}_i), \quad i \notin I_F$<br>$L_S = \frac{L_{S0}+L_{S1}}{2}$	I_F: the index collection of points belonging to features<br>$\hat{p}_i$: predicted feature probability of the i-th point
Center loss (L_C)	$L_{C0} = Avg(-\hat{p}_i^2 \log(1-\hat{p}_i)), \quad i \notin I_F$<br>$L_{Cx} = Avg(-\hat{p}_i^2 \log(1-\hat{p}_i)), \quad i \in I_F,\ i \notin I_C$<br>$L_{C1} = Avg(-p_i(p_i-\hat{p}_i)^2 \log(1-(p_i-\hat{p}_i))),$<br>$i \in I_C$<br>$L_C = \frac{L_{C0}+L_{Cx}+L_{C1}}{3}$	I_F: the index collection of points belonging to features<br>I_C: the index collection of points belonging to feature centers<br>$\hat{p}_i$: predicted center probability of the i-th point<br>p_i: target centrality of the i-th point
Polar mask loss (L_M)	$L_M = Avg\left(\log \frac{\sum_{j=1}^{36} \max(l_{ij}, \hat{l}_{ij})^2}{\sum_{j=1}^{36} \min(l_{ij}, \hat{l}_{ij})^2}\right), \quad i \in I_C$	I_C: the index collection of points belonging to feature centers<br>$\hat{l}_{ij}$: predicted polar mask of the i-th center point<br>l_{ij}: target polar mask of the i-th center point
Total loss (L)	$L = w_S L_S + w_C L_C + w_M L_M$	w_S, w_C, w_M: weights for balancing

Table S2. The accuracy functions of 3D-MSNet. These accuracy functions were used in the calculation of the accuracy curve. Semantic accuracy is the accuracy of the feature semantic prediction branch. Center accuracy is the accuracy of the feature center prediction branch. Polar mask accuracy is the accuracy of the polar mask prediction branch.

Semantic accuracy (ACC_S)	$E_{S0} = Avg(\hat{p}_i), \quad i \in I_F$<br>$E_{S1} = Avg(1 - \hat{p}_i), \quad i \notin I_F$<br>$ACC_S = 1 - \frac{E_{S0} + E_{S1}}{2}$	I_F: the index collection of points belonging to features<br>$\hat{p}_i$: predicted feature probability of the i-th point
Center accuracy (ACC_C)	$E_{C0} = Avg(\hat{p}_i), \quad i \notin I_F$<br>$E_{Cx} = Avg(\hat{p}_i), \quad i \in I_F,\ i \notin I_C$<br>$E_{C1} = Avg(\lvert p_i - \hat{p}_i \rvert), \quad i \in I_C$<br>$ACC_C = 1 - \frac{E_{C0} + E_{Cx} + E_{C1}}{3}$	I_F: the index collection of points belonging to features<br>I_C: the index collection of points belonging to feature centers<br>$\hat{p}_i$: predicted center probability of the i-th point<br>p_i: target centrality of the i-th point
Polar mask accuracy (ACC_M)	$ACC_M = Avg\left(\frac{\sum_{j=1}^{36} \max(l_{ij}, \hat{l}_{ij})}{\sum_{j=1}^{36} \min(l_{ij}, \hat{l}_{ij})}\right), \quad i \in I_C$	I_C: the index collection of points belonging to feature centers<br>$\hat{l}_{ij}$: predicted polar mask of the i-th center point<br>l_{ij}: target polar mask of the i-th center point

Table S3. Optimized parameters used in the TripleTOF 6600 dataset evaluation.

Methods	Step	Parameter	Value
**MarkerView (version 1.3.1)**	Peak Finding Options	Minimum retention time	0.00 min
		Maximum retention time	not check
		Subtraction of # scans	not check
		Minimum spectral peak width	0.01 Da
		Minimum RT peak width	5 scans
		Noise threshold	10
		Assign Charge States	check
	Alignment & Filtering	Retention time tolerance	0.5 min
		Mass tolerance	0.01 Da
		Maximum number of peaks	20000
		Remove peaks in < # samples	4
		Isotope filtering	No filtering
**MS-Dial (version 4.70)**	Data collection	MS1 tolerance	0.015
		MS2 tolerance	0.025
		Retention time begin	0
		Retention time end	100
		MS1 mass range begin	0
		MS1 mass range end	2000
		MS/MS mass range begin	0
		MS/MS mass range end	2000
		Maximun charged number	1
		Consider Cl and Br elements	not check
		Number of threads	1
		Execute retention time correction	not check
	Adduct	[M+H]+	check
		[M+NH4]+	check
		[M+Na]+	check
	Alignment	Reference file	SampleA_1
		Retention time tolerance	0.5
		MS1 tolerance	0.015
		Retention time factor	0.5
		MS1 factor	0.5
		Peak count filter	0
		N% detected in at least one group	0
		Remove features based on blank information	not check
		Gap filling by compulsion	check
	Peak detection	Minimum peak height	1000
		Mass slice width	0.1
		Smoothing method	Linear weighted moving average
		Smoothing level	3
		Minimum peak width	5
		Exclusion mass list	-
**MZmine 2 (version 2.53)**	Raw data methods -> Feature detection -> Mass Detection	Mass detector	Wavelet transform
		Noise level	100
		Scale level	5
		Wavelet window size (%)	60
	Raw data methods -> Feature detection -> ADAP Chromatogram builder	Min group size in # of scans	5
		Group intensity threshold	200
		Min highest intensity	1000
		m/z tolerance	0.015 m/z or 0 ppm
	Feature list methods -> Feature detection -> Chromatogram deconvolution	Algorithm	Baseline cut-off
		m/z center calculation	MEDIAN
		Min peak height	1000
		Peak duration range (min)	0.08 - 10.00
		Baseline level	100
		Amplitude of noise	-
	Feature list methods -> Alignment -> Joint aligner	m/z tolerance	0.015 m/z or 0 ppm
		Weight for m/z	7.5
		Retention time tolerance	0.5 min
		Weight for RT	1
		Require same charge state	not check
		Require same ID	not check
		Compare isotope pattern	not check
		Compare spectra similarity	not check
		Intensity tolerance	100%
		m/z tolerance	0.015 m/z or 0 ppm

	Feature list methods -> Gap filling -> Peak finder(nultithreaded)	Retention time tolerance	0.5 min
**XCMS Online (version 3.7.1)**	Feature Detection	Method<br>ppm<br>minimum peak width<br>maximum peak width<br>mzdiff<br>Signal/Noise threshold<br>Integration method<br>prefilter peaks<br>prefilter intensity<br>Noise filter	centWave<br>5<br>5<br>50<br>0.015<br>5<br>1<br>1<br>200<br>100
	Retention Time Correction	Method<br>profStep	obiwarp<br>1
	Alignment	mzwid<br>bw<br>minfrac<br>minsamp<br>max	0.015<br>5<br>0.5<br>4<br>100
	Annotation	Search for<br>ppm<br>m/z absolute error	isotopes<br>5<br>0.015
**3D-MSNet**	Point cloud extraction	window_mz_width<br>window_rt_width<br>min_intensity<br>from_mz<br>to_mz<br>from_rt<br>to_rt<br>max_peak_mz_width<br>max_peak_rt_width	0.8<br>6<br>128<br>100<br>1300<br>0<br>40<br>0.1<br>1
	Feature detection	experiment<br>epoch<br>mass_analyzer<br>mz_resolution<br>resolution_mz<br>rt_fwhm<br>center_threshold<br>block_rt_width<br>block_mz_width	msnet_20220215_143158<br>300<br>tof<br>35000<br>956<br>0.1<br>0.5<br>6<br>0.8

Table S4. Optimized parameters used in the QE HF dataset evaluation.

Methods	Step	Parameter	Value
**Compound Discoverer (version 3.2.0.421)**	Input Files	-	-
	Select Spectra	Lower RT Limit	0
		Upper RT Limit	0
		Total Intensity Threshold	0
		Minimum Peak Count	1
		Polarity Mode	Is +
		S/N Threshold	1.5
	Align Retention Times	Alignment Fallback	None
		Mass Tolerance	10 ppm
		Maximum Shift [min]	0.3
		Remove Outlier	True
		Shift Reference File	True
		Alignment Model	Adaptive Curve
	Detect Compounds	Ions	M+H
		Intensity Tolerance [%]	30
		Mass Tolerance [ppm]	10 ppm
		Max. Element Counts	C90 H190 Br3 Cl4 K2 N10 Na2 O18 P3 S5
		Min. Element Counts	C H
		Min. Peak Intensity	100000
		S/N Threshold	3
		Filter Peaks	True
		Min. # Isotopes	1
		Min. # Scans per Peak	5
		Max. Peak Width [min]	0.5
		Remove Singlets	False
		Min. Spectral Distance Score	0
		Remove Potentially False Positive Isotopes	True
	Group Compounds	Mass Tolerance	10 ppm
		RT Tolerance [min]	0.5
		Prefered Ions	M+H
	Fill Gaps	Mass Tolerance	10 ppm
		S/N Threshold	1.5
		Use Real Peak Detection	True
**MS-Dial (version 4.70)**	Data collection	MS1 tolerance	0.005
		MS2 tolerance	0.025
		Retention time begin	0
		Retention time end	100
		MS1 mass range begin	0
		MS1 mass range end	2000
		MS/MS mass range begin	0
		MS/MS mass range end	2000
		Maximun charged number	1
		Consider Cl and Br elements	not check
		Number of threads	1
		Execute retention time correction	not check
	Adduct	[M+H]+	check
		[M+NH4]+	check
		[M+Na]+	check
	Alignment	Reference file	SA1
		Retention time tolerance	0.5
		MS1 tolerance	0.005
		Retention time factor	0.5
		MS1 factor	0.5
		Peak count filter	0
		N% detected in at least one group	0
		Remove features based on blank information	not check
		Gap filling by compulsion	check
	Peak detection	Minimum peak height	100000

		Mass slice width	0.1
		Smoothing method	Linear weighted moving average
		Smoothing level	3
		Minimum peak width	5
		Exclusion mass list	-
**MZmine 2 (version 2.53)**	Raw data methods -> Feature detection -> Mass Detection	Mass detector	Wavelet transform
		Noise level	100
		Scale level	5
		Wavelet window size (%)	60
	Raw data methods -> Feature detection -> ADAP Chromatogram builder	Min group size in # of scans	5
		Group intensity threshold	1000
		Min highest intensity	100000
		m/z tolerance	0.005 m/z or 0 ppm
	Feature list methods -> Feature detection -> Chromatogram deconvolution	Algorithm	Noise amplitude
		m/z center calculation	MEDIAN
		Min peak height	100000
		Peak duration range (min)	0.08 - 10.00
		Baseline level	-
		Amplitude of noise	10000
	Feature list methods -> Alignment -> Joint aligner	m/z tolerance	0.005 m/z or 0 ppm
		Weight for m/z	7.5
		Retention time tolerance	0.3 min
		Weight for RT	1
		Require same charge state	not check
		Require same ID	not check
		Compare isotope pattern	not check
		Compare spectra similarity	not check
	Feature list methods -> Gap filling -> Peak finder(nultithreaded)	Intensity tolerance	100%
		m/z tolerance	0.005 m/z or 0 ppm
		Retention time tolerance	0.3 min
**XCMS Online (version 3.7.1)**	Feature Detection	Method	centWave
		ppm	5
		minimum peak width	5
		maximum peak width	50
		mzdiff	0.01
		Signal/Noise threshold	4
		Integration method	1
		prefilter peaks	1
		prefilter intensity	100000
		Noise filter	1000
	Retention Time Correction	Method	obiwarp
		profStep	1
	Alignment	mzwid	0.005
		bw	5
		minfrac	0.5
		minsamp	5
		max	100
	Annotation	Search for	isotopes
		ppm	5
		m/z absolute error	0.015
**3D-MSNet**	Point cloud extraction	window_mz_width	0.4
		window_rt_width	6
		min_intensity	10000
		from_mz	100
		to_mz	1300
		from_rt	0
		to_rt	40
		max_peak_mz_width	0.05
		max_peak_rt_width	1
	Feature detection	experiment	msnet_20220215_143158
		epoch	300
		mass_analyzer	orbitrap
		mz_resolution	60000
		resolution_mz	200
		rt_fwhm	0.08
		center_threshold	0.5

		block_rt_width	6
		block_mz_width	0.4

Table S5. 3D-MSNet parameters used in the Orbitrap XL dataset evaluation.

Methods	Step	Parameter	Value
3D-MSNet	Point cloud extraction	window_mz_width	0.8
		window_rt_width	6
		min_intensity	1000
		from_mz	400
		to_mz	2000
		from_rt	0
		to_rt	120
		max_peak_mz_width	0.2
		max_peak_rt_width	2
	Feature detection	experiment	msnet_20220215_143158
		epoch	300
		mass_analyzer	orbitrap
		mz_resolution	60000
		resolution_mz	400
		rt_fwhm	0.25
		center_threshold	0.6
		block_rt_width	6
		block_mz_width	0.8

Table S6. Match percent of high-confidence features in the Orbitrap XL dataset.

**Samples**	**MaxQuant**	**Dinosaur**	**DeepIso**	**PointIso**	**3D-MSNet**
130124_dilA_1_01	98.37	98.28	79.37	94.65	**98.99**
130124_dilA_1_02	98.16	98.12	71.93	94.64	**98.97**
130124_dilA_1_03	98.56	97.40	82.18	95.61	**98.99**
130124_dilA_1_04	98.55	98.04	74.88	94.40	**98.77**
**Average of sample # 1**	98.41	97.96	77.09	94.83	**98.93**
130124_dilA_2_01	98.32	98.46	80.90	95.75	**98.83**
130124_dilA_2_02	98.04	98.79	73.99	94.17	**98.88**
130124_dilA_2_03	98.06	98.61	71.35	95.54	**98.85**
130124_dilA_2_04	97.74	98.33	72.63	94.18	**98.37**
130124_dilA_2_05	97.72	97.34	72.66	94.87	**98.30**
130124_dilA_2_06	97.83	97.74	74.34	94.18	**98.73**
130124_dilA_2_07	97.95	98.01	72.42	94.46	**98.66**
**Average of sample # 2**	97.95	98.18	74.04	94.74	**98.66**
130124_dilA_3_01	98.73	98.13	72.53	94.80	**99.12**
130124_dilA_3_02	98.54	98.42	81.62	95.06	**99.15**
130124_dilA_3_03	98.17	98.58	72.05	93.80	**99.32**
130124_dilA_3_04	97.99	97.86	80.17	94.20	**98.62**
130124_dilA_3_05	97.89	98.28	71.12	94.62	**99.20**
130124_dilA_3_06	97.89	98.13	72.99	94.05	**98.46**
130124_dilA_3_07	97.88	97.70	81.20	95.37	**98.51**
**Average of sample # 3**	98.16	98.16	75.95	94.56	**98.91**
130124_dilA_4_01	98.60	98.58	82.95	94.61	**99.26**
130124_dilA_4_02	98.28	97.65	80.67	92.67	**99.00**
130124_dilA_4_03	98.67	98.63	71.73	94.54	**99.38**
130124_dilA_4_04	98.25	98.30	81.01	95.79	**99.28**
130124_dilA_4_05	98.05	98.35	71.60	95.84	**99.01**
130124_dilA_4_06	97.23	98.38	79.39	95.88	**98.72**
130124_dilA_4_07	98.16	98.00	81.08	95.49	**99.02**
**Average of sample # 4**	98.18	98.27	78.35	94.97	**99.10**
130124_dilA_5_01	98.46	98.57	74.03	93.72	**98.79**
130124_dilA_5_02	**98.69**	97.92	82.08	93.06	98.60
130124_dilA_5_03	98.02	98.21	72.86	94.30	**99.19**
130124_dilA_5_04	97.70	**98.42**	78.63	95.90	98.39
**Average of sample # 5**	98.22	98.28	76.90	94.25	**98.74**
130124_dilA_6_01	98.58	98.37	82.72	94.22	**98.95**
130124_dilA_6_02	98.32	98.04	77.24	95.56	**99.35**
130124_dilA_6_03	98.51	98.49	72.10	94.41	**99.24**
130124_dilA_6_04	97.54	97.52	69.08	95.34	**98.18**

**Average of sample # 6**	98.24	98.11	75.29	94.88	**98.93**
130124_dilA_7_01	97.87	98.04	81.78	93.93	**98.77**
130124_dilA_7_02	99.05	98.25	68.32	95.76	**99.08**
130124_dilA_7_03	98.16	98.63	68.27	95.40	**98.93**
130124_dilA_7_04	97.41	97.67	81.62	94.99	**98.63**
**Average of sample # 7**	98.12	98.15	75.00	95.02	**98.85**
130124_dilA_8_01	98.20	98.16	81.46	95.07	**98.78**
130124_dilA_8_02	97.91	97.61	81.85	94.02	**98.93**
130124_dilA_8_03	98.34	98.81	71.43	94.14	**99.42**
130124_dilA_8_04	98.34	98.69	81.76	93.79	**99.05**
**Average of sample # 8**	98.20	98.32	79.13	94.26	**99.05**
130124_dilA_9_01	97.99	97.61	81.37	94.04	**98.70**
130124_dilA_9_02	98.35	98.66	72.21	92.92	**99.21**
130124_dilA_9_03	97.61	97.68	82.17	93.97	**98.35**
130124_dilA_9_04	98.16	98.80	81.05	95.40	**99.13**
**Average of sample # 9**	98.03	98.19	79.20	94.08	**98.85**
130124_dilA_10_01	98.05	97.58	68.17	94.34	**98.98**
130124_dilA_10_02	98.42	98.30	66.67	89.96	**99.35**
130124_dilA_10_03	97.92	97.69	72.69	94.46	**99.07**
130124_dilA_10_04	98.66	98.56	80.52	94.31	**99.26**
**Average of sample # 10**	98.26	98.03	72.01	93.27	**99.17**
130124_dilA_11_01	98.06	97.71	73.44	94.17	**98.83**
130124_dilA_11_02	98.56	98.31	74.15	90.44	**99.08**
130124_dilA_11_03	97.86	97.35	83.85	92.61	**98.48**
130124_dilA_11_04	97.67	97.81	82.46	95.25	**98.91**
**Average of sample # 11**	98.04	97.80	78.48	93.12	**98.83**
130124_dilA_12_01	**98.28**	96.87	76.81	89.71	97.87
130124_dilA_12_02	97.72	97.29	75.10	90.68	**98.26**
130124_dilA_12_03	97.18	96.89	76.07	91.41	**97.64**
130124_dilA_12_04	**97.67**	97.18	82.14	92.66	97.28
**Average of sample # 12**	97.71	97.06	77.53	91.12	**97.76**
**Average of all samples**	98.12	98.07	76.51	94.19	**98.83**

Table S7. Match number of high-confidence features in the Orbitrap XL dataset.

Samples	MaxQuant	MaxQuant	Dinosaur	DeepIso	PointIso	3D-MSNet
130124_dilA_1_01	4246	4177	4173	3370	4019	**4203**
130124_dilA_1_02	4735	4648	4646	3406	4481	**4686**
130124_dilA_1_03	4736	4668	4613	3892	4528	**4688**
130124_dilA_1_04	4889	4818	4793	3661	4615	**4829**
130124_dilA_2_01	5722	5626	5634	4629	5479	**5655**
130124_dilA_2_02	5459	5352	5393	4039	5141	**5398**
130124_dilA_2_03	5990	5874	5907	4274	5723	**5921**
130124_dilA_2_04	5878	5745	5780	4269	5536	**5782**
130124_dilA_2_05	5834	5701	5679	4239	5535	**5735**
130124_dilA_2_06	5755	5630	5625	4278	5420	**5682**
130124_dilA_2_07	5815	5696	5699	4211	5493	**5737**
130124_dilA_3_01	6040	5963	5927	4381	5726	**5987**
130124_dilA_3_02	6007	5919	5912	4903	5710	**5956**
130124_dilA_3_03	6064	5953	5978	4369	5688	**6023**
130124_dilA_3_04	6227	6102	6094	4992	5866	**6141**
130124_dilA_3_05	6398	6263	6288	4550	6054	**6347**
130124_dilA_3_06	6101	5972	5987	4453	5738	**6007**
130124_dilA_3_07	6261	6128	6117	5084	5971	**6168**
130124_dilA_4_01	5700	5620	5619	4728	5393	**5658**
130124_dilA_4_02	5624	5527	5492	4537	5212	**5568**
130124_dilA_4_03	5769	5692	5690	4138	5454	**5733**
130124_dilA_4_04	6004	5899	5902	4864	5751	**5961**
130124_dilA_4_05	6243	6121	6140	4470	5983	**6181**
130124_dilA_4_06	6095	5926	5996	4839	5844	**6017**
130124_dilA_4_07	6206	6092	6082	5032	5926	**6145**
130124_dilA_5_01	5380	5297	5303	3983	5042	**5315**
130124_dilA_5_02	5782	**5706**	5662	4746	5381	5701
130124_dilA_5_03	5962	5844	5855	4344	5622	**5914**
130124_dilA_5_04	6093	5953	**5997**	4791	5843	5995
130124_dilA_6_01	5342	5266	5255	4419	5033	**5286**
130124_dilA_6_02	5830	5732	5716	4503	5571	**5792**
130124_dilA_6_03	5777	5691	5690	4165	5454	**5733**
130124_dilA_6_04	6051	5902	5901	4180	5769	**5941**
130124_dilA_7_01	5861	5736	5746	4793	5505	**5789**
130124_dilA_7_02	6184	6125	6076	4225	5922	**6127**
130124_dilA_7_03	6429	6311	6341	4389	6133	**6360**
130124_dilA_7_04	6131	5972	5988	5004	5824	**6047**
130124_dilA_8_01	5826	5721	5719	4746	5539	**5755**

130124_dilA_8_02	5702	5583	5566	4667	5361	**5641**
130124_dilA_8_03	5733	5638	5665	4095	5397	**5700**
130124_dilA_8_04	5977	5878	5899	4887	5606	**5920**
130124_dilA_9_01	5318	5211	5191	4327	5001	**5249**
130124_dilA_9_02	5452	5362	5379	3937	5066	**5409**
130124_dilA_9_03	5519	5387	5391	4535	5186	**5428**
130124_dilA_9_04	5658	5554	5590	4586	5398	**5609**
130124_dilA_10_01	4914	4818	4795	3350	4636	**4864**
130124_dilA_10_02	4165	4099	4094	2777	3747	**4138**
130124_dilA_10_03	5378	5266	5254	3909	5080	**5328**
130124_dilA_10_04	5540	5466	5460	4461	5225	**5499**
130124_dilA_11_01	4371	4286	4271	3210	4116	**4320**
130124_dilA_11_02	4016	3958	3948	2978	3632	**3979**
130124_dilA_11_03	4341	4248	4226	3640	4020	**4275**
130124_dilA_11_04	4756	4645	4652	3922	4530	**4704**
130124_dilA_12_01	2906	**2856**	2815	2232	2607	2844
130124_dilA_12_02	2984	2916	2903	2241	2706	**2932**
130124_dilA_12_03	3051	2965	2956	2321	2789	**2979**
130124_dilA_12_04	3052	**2981**	2966	2507	2828	2969

Table S8. Average multi-match number of high-confidence features in the Orbitrap XL dataset.

In the matching of the high-confidence features in feature detection results, each high-confidence feature may match more than one detected feature, which is often attributed to duplicate or erroneous feature extractions. 3D-MSNet achieved the best feature extraction accuracy in comparison with other software with the lowest multi-match ratio in most samples (56 of 57) and the lowest average multi-match ratio in all 12 samples.

**Samples**	**MaxQuant**	**Dinosaur**	**DeepIso**	**PointIso**	**3D-MSNet**
130124_dilA_1_01	1.079	1.004	1.016	4.674	**1.001**
130124_dilA_1_02	1.094	1.012	1.025	4.694	**1.001**
130124_dilA_1_03	1.100	1.009	1.026	4.674	**1.004**
130124_dilA_1_04	1.081	1.005	1.014	4.632	**1.002**
**Average of sample # 1**	1.088	1.007	1.020	4.669	**1.002**
130124_dilA_2_01	1.082	1.011	1.013	4.674	**1.002**
130124_dilA_2_02	1.092	1.007	1.014	4.547	**1.003**
130124_dilA_2_03	1.089	1.013	1.015	4.635	**1.003**
130124_dilA_2_04	1.082	1.007	1.012	4.451	**1.002**
130124_dilA_2_05	1.088	1.012	1.010	4.640	**1.004**
130124_dilA_2_06	1.080	1.007	1.012	4.489	**1.005**
130124_dilA_2_07	1.086	1.010	1.012	4.582	**1.002**
**Average of sample # 2**	1.086	1.010	1.012	4.574	**1.003**
130124_dilA_3_01	1.076	1.008	1.011	4.492	**1.001**
130124_dilA_3_02	1.081	1.009	1.016	4.577	**1.004**
130124_dilA_3_03	1.085	1.004	1.022	4.447	**1.002**
130124_dilA_3_04	1.087	1.005	1.010	4.572	**1.004**
130124_dilA_3_05	1.083	1.006	1.018	4.582	**1.005**
130124_dilA_3_06	1.095	1.005	1.009	4.611	**1.002**
130124_dilA_3_07	1.077	1.009	1.016	4.559	**1.001**
**Average of sample # 3**	1.083	1.007	1.015	4.549	**1.003**
130124_dilA_4_01	1.076	1.007	1.015	4.583	**1.000**
130124_dilA_4_02	1.073	1.009	1.018	4.257	**1.005**
130124_dilA_4_03	1.077	1.008	1.017	4.388	**1.002**
130124_dilA_4_04	1.085	1.005	1.014	4.665	**1.003**
130124_dilA_4_05	1.093	1.006	1.013	4.627	**1.004**
130124_dilA_4_06	1.081	1.007	1.016	4.588	**1.004**
130124_dilA_4_07	1.100	1.009	1.013	4.444	**1.001**
**Average of sample # 4**	1.084	1.007	1.015	4.507	**1.003**
130124_dilA_5_01	1.067	1.011	1.013	4.443	**1.001**
130124_dilA_5_02	1.083	1.005	1.012	4.614	**1.004**
130124_dilA_5_03	1.074	1.005	1.008	4.360	**1.002**
130124_dilA_5_04	1.076	1.008	1.012	4.584	**1.002**
**Average of sample # 5**	1.075	1.007	1.011	4.500	**1.002**

130124_dilA_6_01	1.067	1.008	1.015	4.459	**1.002**
130124_dilA_6_02	1.081	1.012	1.010	4.773	**1.004**
130124_dilA_6_03	1.076	1.010	1.011	4.444	**1.003**
130124_dilA_6_04	1.086	1.008	1.009	4.608	**1.004**
**Average of sample # 6**	1.077	1.009	1.011	4.571	**1.003**
130124_dilA_7_01	1.066	1.005	1.014	4.504	**1.001**
130124_dilA_7_02	1.079	1.005	1.010	4.737	**1.003**
130124_dilA_7_03	1.076	1.006	1.012	4.589	**1.002**
130124_dilA_7_04	1.079	1.006	1.023	4.516	**1.003**
**Average of sample # 7**	1.075	1.005	1.015	4.587	**1.002**
130124_dilA_8_01	1.071	1.011	1.012	4.735	**1.001**
130124_dilA_8_02	1.084	1.011	1.016	4.505	**1.005**
130124_dilA_8_03	1.064	1.004	1.012	4.366	**1.002**
130124_dilA_8_04	1.085	1.009	1.011	4.335	**1.003**
**Average of sample # 8**	1.076	1.009	1.013	4.485	**1.003**
130124_dilA_9_01	1.072	1.011	1.012	4.573	**1.000**
130124_dilA_9_02	1.082	1.007	1.017	4.522	**1.005**
130124_dilA_9_03	1.091	**1.004**	1.022	4.532	1.005
130124_dilA_9_04	1.082	1.007	1.016	4.333	**1.002**
**Average of sample # 9**	1.082	1.007	1.017	4.490	**1.003**
130124_dilA_10_01	1.079	1.007	1.012	4.551	**1.003**
130124_dilA_10_02	1.077	1.007	1.022	4.494	**1.001**
130124_dilA_10_03	1.095	1.006	1.014	4.431	**1.001**
130124_dilA_10_04	1.081	1.007	1.014	4.494	**1.001**
**Average of sample # 10**	1.083	1.007	1.015	4.492	**1.001**
130124_dilA_11_01	1.091	1.008	1.021	4.430	**1.001**
130124_dilA_11_02	1.080	1.012	1.018	4.339	**1.002**
130124_dilA_11_03	1.079	1.011	1.014	4.922	**1.002**
130124_dilA_11_04	1.098	1.011	1.017	4.564	**1.001**
**Average of sample # 11**	1.087	1.010	1.017	4.564	**1.002**
130124_dilA_12_01	1.087	1.009	1.011	4.575	**1.000**
130124_dilA_12_02	1.098	1.006	1.018	4.363	**1.003**
130124_dilA_12_03	1.097	1.008	1.022	4.580	**1.001**
130124_dilA_12_04	1.100	1.009	1.008	4.664	**1.000**
**Average of sample # 12**	1.095	1.008	1.014	4.545	**1.001**
**Average of all samples**	1.083	1.008	1.015	4.544	**1.002**

Table S9. Time cost on different evaluation datasets. Total analysis time is the time cost of all files. Average analysis time is the average time cost per file.

		Total analysis time (min)	Average analysis time (min)
TripleTOF 6600 dataset (8 files)	MarkerView	0.8	0.1
	MS-DIAL	16.4	2.1
	MZmine 2	30.9	3.9
	XCMS Online	574.7	71.8
	**3D-MSNet* (Ours)**	37.4	4.7
QE HF dataset (10 files)	Compound Discoverer	351	35.1
	MS-DIAL	46.1	4.6
	MZmine 2	59.4	5.9
	XCMS Online	879.5	88.0
	**3D-MSNet* (Ours)**	187.6	18.8
Orbitrap XL dataset (57 files)	MaxQuant	1044.9	18.3
	Dinosaur	41.7	0.7
	DeepIso*	11281.6	197.9
	PointIso*	6829.4	119.8
	**3D-MSNet* (Ours)**	1207.2	21.2

The methods marked with * are based on deep learning.

Table S10. Evaluation of proteomics software on the metabolomics datasets

To perform comprehensive comparisons of 3D-MSNet, we also evaluated proteomics analysis software on the metabolomics dataset. However, the proteomics analysis software did not achieve competitive analysis results. Since the data distributions were different between proteomics and metabolomics datasets, we believed that it was unfair to present their inferiority on metabolomics datasets in the main text.

The results were summarized below for readers' reference, and it can also show the value of 3D-MSNet that can support the analysis of metabolomics and proteomics data sets at the same time.

The specific results are shown in the table below. Proteomics software (MaxQuant, Dinosaur, DeepIso, PointIso) did not perform well on the metabolomics benchmark datasets. MaxQuant and Dinosaur had low feature detection rate. DeepIso and PointIso were deep learning based models, sensitive to data distributions and produced unreliable results.

TripleTOF 6600 dataset

	Matched features	Accurately quantified features	Quantification accuracy (%)	Accurately quantified rate (%)	True markers	False markers	Marker detection accuracy (%)
MarkerView	911	880	96.6	90.7	73	9	89.0
MS-DIAL	891	754	84.6	77.7	71	99	41.8
MZmine2	958	884	92.3	91.1	82	26	75.9
XCMS Online	948	583	61.5	60.1	80	262	23.4
**3D-MSNet (Ours)**	**938**	**928**	**98.9**	**95.7**	**86**	**0**	**100.0**
MaxQuant	735	716	97.4	73.8	58	5	92.1
Dinosaur	852	762	89.4	78.6	79	26	75.2
DeepIso	491	358	72.9	36.9	13	44	22.8
PointIso	657	281	42.8	29.0	55	179	23.5

QE HF dataset

	Matched features	Accurately quantified features	Quantification accuracy (%)	Accurately quantified rate (%)	True markers	False markers	Marker detection accuracy (%)
Compound Discoverer	771	720	93.4	74.2	60	29	67.4
MS-DIAL	784	663	84.6	68.4	63	19	76.8
MZmine2	806	783	97.1	80.7	61	4	93.8
XCMS Online	820	747	91.1	77.0	64	18	78.0
**3D-MSNet (Ours)**	**823**	**820**	**99.6**	**84.5**	**65**	**0**	**100.0**
MaxQuant	686	682	99.4	70.3	56	0	100.0
Dinosaur	792	786	99.2	81.0	61	1	98.4
DeepIso	639	551	86.2	56.8	48	11	81.4
PointIso	298	130	43.6	13.4	20	66	23.3

Table S11. Differences between 3D-MSNet and PointIso.

Considering that readers will be interested in the difference between the two point-cloud-based deep learning methods, 3D-MSNet and PointIso, we present the difference comparison table below. 3D-MSNet and PointIso were developed with different development intentions.

	PointIso	3D-MSNet
Intention of development	Solve the problems of low data accuracy, large data volume, large number of blank pixels in the process of converting MS1 data into images in the DeepIso model. Run faster and support 4D data analysis.	Perform more accurate feature detection, quantification, and overlapping feature separation by fully utilizing and analyzing the local spatial distribution of all MS1 signal points.
Targeted data type	3D and 4D LC-MS map of proteomics data.	3D LC-MS map of metabolomics and proteomics data.
Purpose of using neural network	Detect multi-isotopic pattern in LC-MS maps.	Detect, quantify 3D peaks and distinguish overlapping 3D peaks in LC-MS maps.
Analysis precision	With precision loss. Although PointIso is described as "arbitrary-precision" in the paper, the accuracy is lost in multiple places. For example, when converting mass spectrum data into point clouds, the data is still gridded and filtered, only the signal points with the highest intensity in each "arbitrary size" bin are retained; when the number of data points in the point cloud is greater than 5000, the excess will be discarded.	No precision loss. In the data splitting step, all data points and their original precision are preserved. The network supports dynamic point input number, and no points are discarded during analysis.
Data preprocessing	PointIso follows the processing method of DeepIso and normalizes the intensity ratio to the range of 0-255	3D-MSNet takes the logarithm of the signal intensity, which can significantly improve the feature detection and quantification accuracy of low-signal peaks. 3D-MSNet standardizes the RT and m/z dimensions of the point cloud according to the RT full-width at half maximum (FWHM) and the m/z FWHM, which is calculated at different m/z according to the principle of mass spectrometry.
Point cloud block segmentation	PointIso performs attention based scanning of LC-MS map through completely non-overlapping window. In the analysis of each window, nine windows centered on the current window are analyzed at the same time in certain steps, which is equivalent to a considerable boundary that needs repeated analysis.	3D-MSNet segments point clouds with overlapping windows. 3D-MSNet uses overlapping windows (extended windows) in feature detection to ensure the integrity of the feature at the segmentation boundary, and uses non-overlapping windows (initial windows) when merging results to ensure that the results are not duplicated.

Network design	PointNet is a multi-stage deep-learning-based model. In the IsoDetecting stage, PointNet performs semantic segmentation for point clouds to predict charge state and extract spatial position of isotopes. PointIso uses PointNet to extract local and global features. Due to the limitations of the analysis principle, PointNet requires a fixed number of input points, has weak local feature extraction capabilities, and is sensitive to the data scale. In addition, PointIso inherits the T-Net module in PointNet, which is used to eliminate the influence of affine transformation of point clouds, but the elimination of the direction will make the model unable to use the unique distribution characteristics of mass spectrometry data in the m/z and RT directions. In IsoGrouping stage, point clouds are converted to low-resolution image and analysed by a image-based network to group isotopes into multi-isotope pattern. In a multi-stage model, the errors of each stage model will accumulate and affect the final result.	3D-MSNet is a single-stage multi-task deep-learning-based model. 3D-MSNet directly finds three-dimensional features (peaks) instead of isotope patterns. 3D-MSNet supports the analysis of point clouds with arbitrary number of points without loss of precision. 3D-MSNet composed of a feature extraction backbone, three prediction branches, and a prediction result assembly mechanism. In the feature extraction backbone, 3D-MSNet first extracts local spatial features of each point with Local Spatial Encoding block, which is similar to PointNet++ (different from PointNet) and with additional density information. Then 3D-MSNet applies multi-layer encoding blocks and decoding blocks, and uses skip connections to associate the features of the corresponding layers to obtain local-global features of different scales and levels. To better grasp the spatial information of each point, 3D-MSNet encodes the spatial position and density of neighboring points in Local Spatial Encoding block, and uses InterpConv in Encoding Block to further improve the model's sense of direction and distance in m/z and RT dimensions. In the three prediction branches, 3D-MSNet uses the features extracted by backbone to perform feature semantic prediction, feature center prediction, and feature boundary prediction. Then, the predicted results of the three branches are unified into final results by prediction result assembly mechanism. The errors of each branch are mutually corrected in this process.
Feature boundary determination	The boundaries of isotope patterns are determined by the IsoGrouping network. The boundaries of each detected isotope are determined by fixed m/z and RT tolerance. The intensities of the isotopes are quantified by summing the intensity values of all points within a fixed window of the downsampled point clouds.	3D-MSNet predicts a polar mask for each feature to obtain more robust quantification results, which is a 2D feature contour on the m/z and RT plane represented by a center point and multiple equiangular rays in Polar Coordinates. The intensities of the features are computed from the volume of the 3D features within the predicted boundaries.
Training method	PointIso believes that human annotation of peptide features is out of bounds due to the gigapixel size of LC-MS plots. PointIso was	3D-MSNet was trained on the self-annotated 3DMS dataset. Each signal point in the dataset is manually assigned an instance label indicating

	trained on the intersection of feature lists generated by MaxQuant and Dinosaur.	whether the point belongs to a certain feature and to which feature it belongs.

Appendix S1. Marker selection criteria on the metabolomics datasets.

For differential marker selection from metabolomics data, we changed the original criteria in the benchmark study (FC < 0.5 or FC > 2) and replaced it by "consensus features whose fold-changes were out of 20% tolerance of the range of (0.5, 2)".

Although the original criteria are widely used for marker selection, it is a compromise method to help initial screening of potential markers when we do not know the distribution of relative quantitative results. In this study, we know that there are two groups of compounds (Gd3, Gd4) which have quantification fold-changes exactly equal to 0.5 and 2. Following the original criteria, due to the bias caused by sample preparation and LC-MS acquisition, half of the compounds in these two groups will be rigidly classified into "non-markers", and the number of markers can no longer be used as a measure of software performance. For example, suppose there are 30 compounds in Gd4, and the ground-truth fold-changes are distributed between 1.8 and 2.2. Accounting for bias in LC-MS acquisitions, we assume that 10 compounds have a fold-change less than 2 and 20 compounds have a fold-change greater than 2. Through original criteria screening, software A detects 18 markers, and software B detects 22 markers. Then, what can be inferred from the marker numbers? Is software B better than software A? We cannot tell. Using the original criteria, quantization bias of the LC-MS system deprives the number of markers as a measure of software accuracy. But if we add a relaxation to the criteria, for example, adjust the threshold to 1.7, outside the bias distribution interval, assuming that software A detects 27 markers and software B detects 29 markers, then the number of markers can clearly reflect that B is better than A.

Considering that the commonly used quantification deviation is 20%, and we also used 20% tolerance for accurate quantified feature selection, we added a 20% relaxation to the original criteria $[0,\ 2^{-1}]$ || $[2^{1}, +\infty)$, and got the criteria used in this study $[0,\ 2^{-(1-0.2)}]$ || $[2^{1-0.2}, +\infty)$. Using the relaxed criteria, the number of markers can be directly used as a parameter to measure the accuracy of the software. The relaxed criteria have greater meaning for software comparisons than the original criteria.

Appendix S2. Feature matching method in evaluation of the proteomics Orbitrap XL dataset.

In comparison of the proteomics dataset, we matched the feature detection results of different software with the high confidence identifications of MASCOT. When the feature detection result only has center RT and m/z information, we performed matching according to m/z and RT tolerance. When the feature detection result contains the m/z and RT distribution range information of the feature, we filtered again based on the information, and only kept the feature that contained the MASCOT result in the feature m/z and RT range. Because the high confidence identifications were obtained by scoring of MS2 spectra, we believed that only the features that contains the precursor m/z and RT of the MS2 spectra were correct matches.

In the result matching of PointIso, the detection ratio for PointIso (94.19%) in Fig. 5a of this study is not consistent with that reported by PointIso itself ("an average detection rate of PointIso is 98.01% across 12 samples"). With the filtering method described above, before filtering by RT range, PointIso indeed achieved a detection rate of 98%, and after filtering, it turned to about 94% as shown in the paper. In the evaluation of the proteomics software, only MaxQuant was exempted from RT range filtering, since we did not find RT range information in its result table.

Appendix S3. Evaluation on the Orbitrap XL dataset with the targeted peptide library.

1. Dataset

The Orbitrap XL dataset contains 12 samples at different dilution levels. Each sample has 4 to 7 replicate injections and is composed by synthetic potato peptides, synthetic human peptides, and non-variable *Streptococcus pyogenes* strain SF370 background peptides. The dilution levels are listed in the following table.

Table A3.1. Dilution levels of the Orbitrap XL dataset.

Sample	Replicates	Potato Relative Concentration	Human Relative Concentration	Comparison	Potato FC	Human FC
1	4	1	0.00001			
2	7	0.33	0.000033	2 vs 1	3.33	3.30
3	7	0.12	0.000067	3 vs 2	2.75	2.03
4	7	0.1	0.0001	4 vs 3	1.20	1.49
5	4	0.033	0.00033	5 vs 4	3.03	3.30
6	4	0.01	0.001	6 vs 5	3.30	3.03
7	4	0.0033	0.0033	7 vs 6	3.03	3.30
8	4	0.001	0.01	8 vs 7	3.30	3.03
9	4	0.00033	0.033	9 vs 8	3.03	3.30
10	4	0.0001	0.1	10 vs 9	3.30	3.03
11	4	0.000033	0.33	11 vs 10	3.03	3.30
12	4	0.00001	1	12 vs 11	3.30	3.03

2. Library

The Orbitrap XL dataset did not provide a spike-in and background peptide library with peptide m/z and RT in its paper and project FTP. However, the Orbitrap XL dataset has a twin targeted dataset, which was acquired with targeted SRM (selected reaction monitoring) method using the same LC platform. The SRM dataset has a peptide library, which contains 47 synthetic potato peptides, 78 synthetic human peptides, and 29 background peptides.

To evaluate the feature detection and quantification performance on the Orbitrap XL dataset, we tried to use the targeted SRM library for result matching.

3. Alignment

In the evaluations of the metabolomics datasets, we did not introduce additional alignment methods, since the datasets has less injections (8 and 10), and almost no bias in RT. However, the Orbitrap XL dataset has 57 runs. RT shifts obviously so that we cannot ignore it.

We performed alignment by G-Aligner, a self-developed hybrid non-centric alignment method, which first performs coarse RT warping and then performs fine assignment based on graph theory and discrete optimization to achieve feature-to-feature alignment. The alignment procedure was performed in two steps. In the first step, we aligned the replicates for each of the 12 samples. In the second step, we aligned the 12 samples based on the median m/z and RT of the aligned replicates and assembled the alignment results. We used the same set of parameter on all software results.

4. Feature matching

To find features corresponding to the peptide library, we searched feature extraction results with a settled searching window (0.01Da m/z tolerance, 2min RT tolerance). The m/z tolerance was the same as its used in MASCOT high-confidence feature library evaluation. The RT tolerance was the same as the tolerance set in the targeted SRM acquisition, which was the narrowest RT range we could find from the dataset. In our experiments, the number of matches had almost no change when using larger RT tolerance.

The matched features should not only be detected in the matching window, but also follow the distribution trend as the theoretical dilution concentrations. In the trend estimation step, trend of each peptide was estimated by least-square linear regression on part of the results, potato peptides on results of sample 1 to 6, human peptides on sample 7 to 12, and background peptides on all samples. The unselected part of results had low intensity, hard to detect, and prone to noise.

As for multi-matched peptide selection, the first priority was the number of detected features in all injections, and the second priority was high intensity.

5. Normalization

In the evaluation of the metabolomics datasets, we did not introduce additional normalization methods since the quantification results were stable. However, the intensity varies obviously in the Orbitrap XL dataset, even among the replicates of the same sample.

To eliminate the intensity deviation caused by pretreatment and LC-MS system, we estimated the intensity trend of background peptides among injections. The trend estimation was performed in two steps. In the first step, we calculated the trend for each background peptide, which was the quantitative results across all samples of the current peptide divided by the median. In the second step, the trend was estimated by calculating the median of the background peptide trends.

The estimated trends of compared software are shown in Figure A3.2. The trends of MaxQuant, Dinosaur, and 3D-MSNet were highly similar, proving the high quantification accuracy of these software. The trend of PointIso was less similar, which was caused by less accurate quantification (Figure A3.3, A3.5, A3.7). The trend of DeepIso was different from MaxQuant, and the results of DeepIso could only be normalized with its own trend, which might be caused by different quantification strategy. If normalized by other trends, for example, the trend of MaxQuant, the quantification results showed larger deviations in replicates.

Figure A3.2: Estimated normalization trends of the Orbitrap XL dataset.

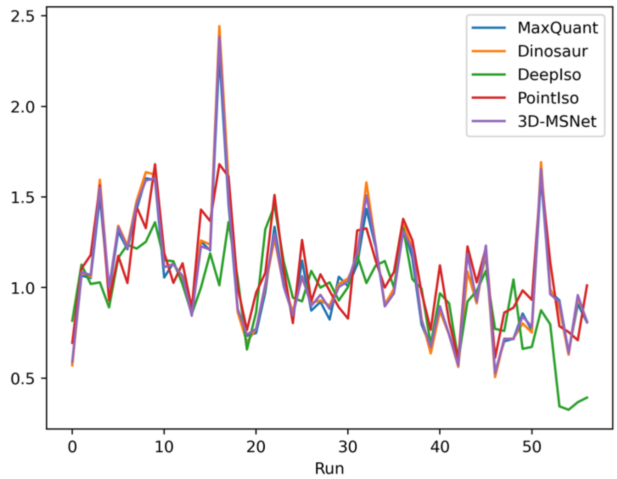

We normalized software with their trends and present the results in Figure A3.3. Each colored line represents the intensity change of a matched peptide in different samples.

Figure A3.3: Quantification results before and after normalization (a. Results of synthetic potato peptides. b. Results of synthetic human peptides. c. Results of background peptides).

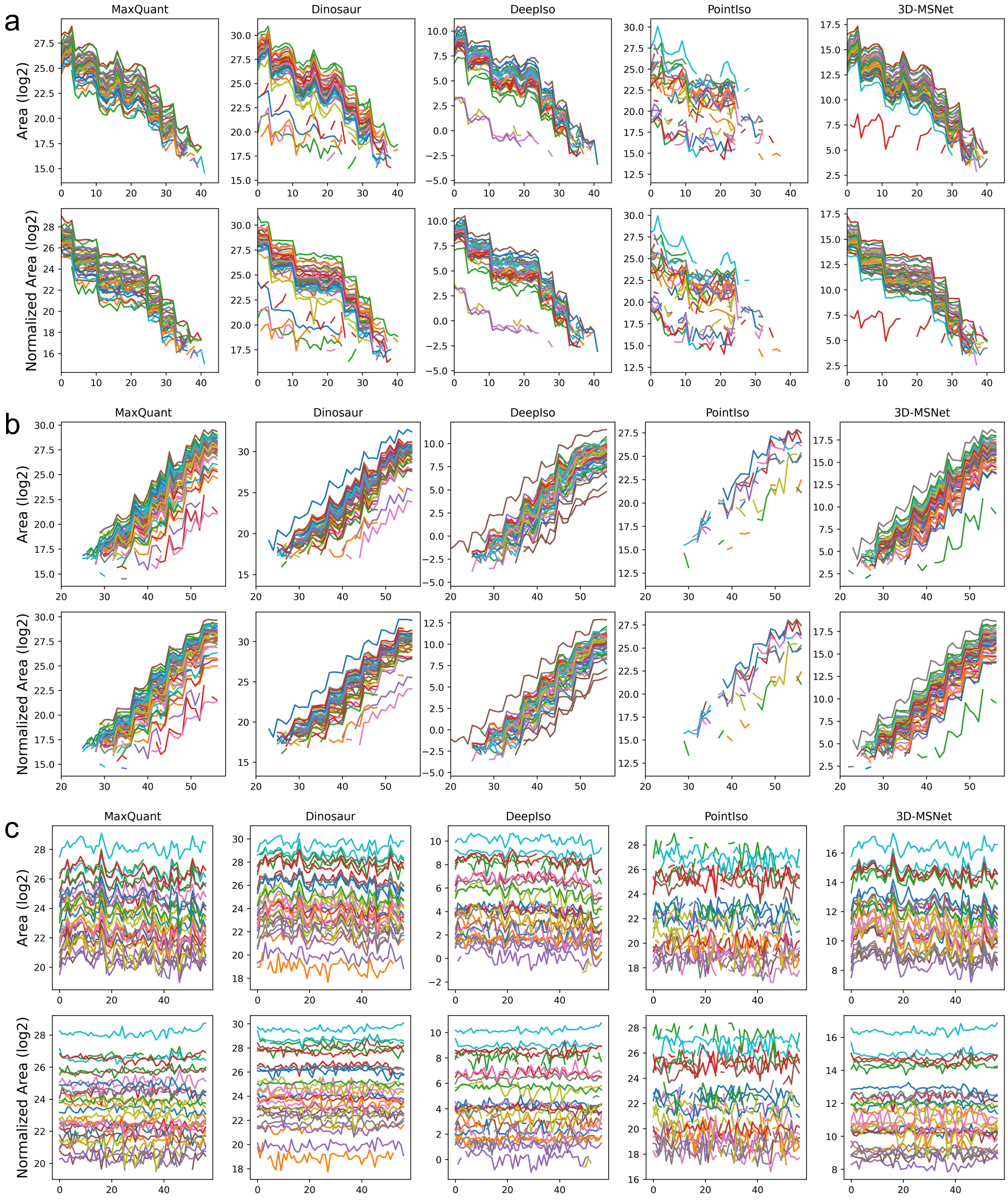

As shown in Figure A3.3, MaxQuant, Dinosaur, DeepIso, and 3D-MSNet showed results that complied with the distribution of the dilution levels. PointIso was significantly lower than other methods in number of matches and quantitative accuracy. PointIso had low quantification accuracy, resulting in few results that met the matching rules (see Appendix S1 4. Feature matching). Considered together with the high false positive rate (see Table S8), we believed that the current version of PointIso still needed more tuning.

6. Feature detection result evaluation

The matched results are presented in Table A3.4. The targeted library contains 47 potato peptides, 78 human peptides, and 29 background peptides. Correspondingly, there are 2679 potato features, 4446 human features, and 1653 background features to match in 57 injections. Since the peptides in the potato and human sets had very low concentrations in part of the samples, the feature detection ratio of these two sets was relatively low.

3D-MSNet achieved the highest feature detection rate and average number of features per peptide on the potato set and the human set, slightly lower than Dinosaur on the background set. Overall, among the three sets, 3D-MSNet achieved the highest feature detection rate. The feature detection rate can be ordered by the number of matched features: 3D-MSNet > MaxQuant > Dinosaur > DeepIso > PointIso. Comparing to the feature detection rate presented in Fig.5a, the detection rate rankings of DeepIso and PointIso are different. This is because we screened out quantitatively biased results in feature matching (see Appendix S1 4. Feature matching). PointIso showed lower quantification accuracy, which resulted to declines in the matching rates.

Table A3.4: Feature detection results based on the SRM library.

		Matched features (57 runs)	Feature detection rate (%)	Matched peptides	Peptide detection rate (%)	Features per matched peptide
Potato set	Library	2679	-	47	-	-
	MaxQuant	1416	52.9	43	91.5	32.9
	Dinosaur	1341	50.1	42	89.4	31.9
	DeepIso	1138	42.5	35	74.5	32.5
	PointIso	660	24.6	35	74.5	18.9
	**3D-MSNet (Ours)**	**1502**	**56.1**	**44**	**93.6**	**34.1**
Human set	Library	4446	-	78	-	-
	MaxQuant	1463	32.9	**60**	**76.9**	24.4
	Dinosaur	1191	26.8	46	59.0	25.9
	DeepIso	1288	29.0	50	64.1	25.8
	PointIso	179	4.0	11	14.1	16.3
	**3D-MSNet (Ours)**	**1523**	**34.3**	56	71.8	**27.2**
Background set	Library	1653	-	29	-	-
	MaxQuant	1606	97.2	29	100.0	55.4
	Dinosaur	**1630**	**98.6**	29	100.0	**56.2**
	DeepIso	1517	91.8	29	100.0	52.3
	PointIso	1224	74.0	29	100.0	42.2
	**3D-MSNet (Ours)**	1625	98.3	29	100.0	56.0

7. Feature quantification result evaluation

To evaluate the stability of quantification, we calculated the coefficient of variance (CV) values of normalized quantification results of matched features among replicates. The distributions of CV values are shown in Figure A3.5. Each point represents the CV value of the quantitative results of a peptide in all replicates of the current sample. Since the low-concentration features that are rarely detected will bring statistical errors, we only need to pay attention to the distribution of samples with more feature detection (Potato set: sample 1 to 7, Human set: sample 5 to 12, Background set: all samples).

Figure A3.5: Distribution of CV values (a. Results of synthetic potato peptides. b. Results of synthetic human peptides. c. Results of background peptides).

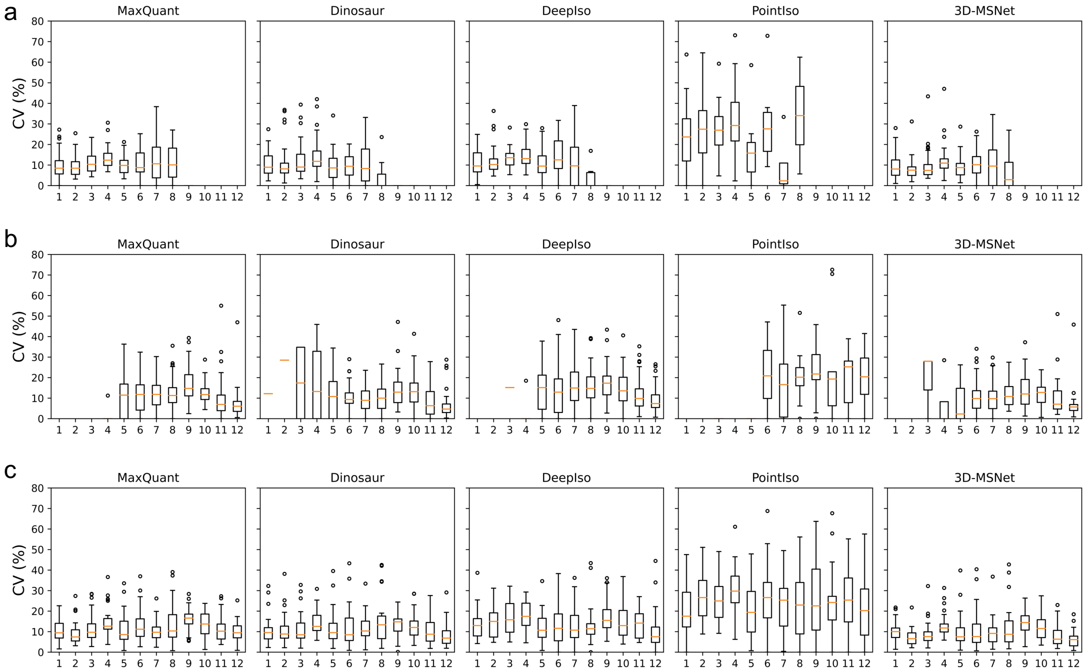

As shown in Figure A3.5, MaxQuant, Dinosaur, DeepIso, and 3D-MSNet had similar distributions. DeepIso had higher CV values. For a clearer quantitative evaluation, we calculated the mean of the CV means among samples (Potato set: sample 1 to 7, Human set: sample 5 to 12, Background set: all samples). The calculated results are summarized in the Table A3.6. 3D-MSNet achieved the lowest CV value in all the three sets, which represented that 3D-MSNet had the highest quantification stability. The stability ranking is summarized as follows: 3D-MSNet > Dinosaur > MaxQuant > DeepIso > PointIso.

Table A3.6: Average CV (%) values.

	Potato set	Human set	Background set
MaxQuant	11.0	11.6	12.2
Dinosaur	11.1	10.6	12.0
DeepIso	12.5	14.1	14.3
PointIso	23.8	19.2	24.7
**3D-MSNet (Ours)**	**9.9**	**10.2**	**10.3**

To evaluate the accuracy of the quantification, we calculated the mean area in replicates to obtain the quantitative results of each matched peptide in each group. Then, we divided areas between adjacent samples to fold changes (FC) and compared the fold changes with the theoretical value in Table A3.1.

For each measured fold change, we divided the fold change by theoretical concentration ratio to obtain FC ratio. The distributions of FC Ratios are shown in Figure A3.7. Each point in the figure represents the measured FC ratio of a peptide in two adjacent samples. The closer to 1, the more consistent the quantitative result is with the theoretical concentration ratio. Since the low-concentration features that are rarely detected will bring statistical errors, we only need to pay attention to the distribution of samples with more feature detection (Potato set: sample 1 to 7, Human set: sample 5 to 12, Background set: all samples).

Figure A3.7: Distribution of FC ratios (a. Results of synthetic potato peptides. b. Results of synthetic human peptides. c. Results of background peptides).

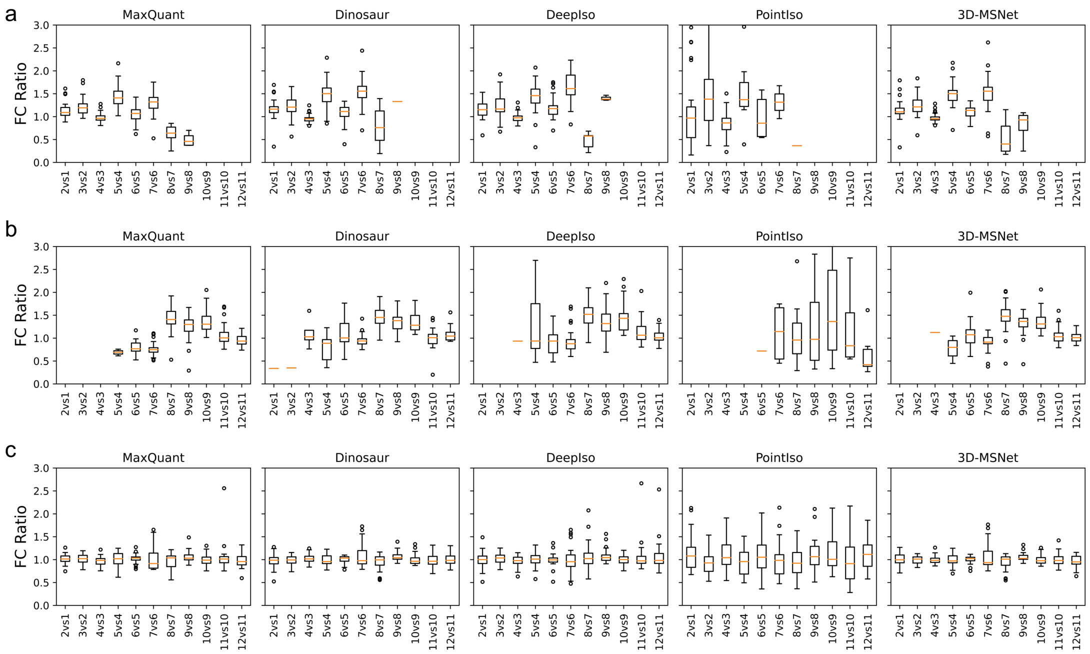

As shown in Figure A3.7, MaxQuant, Dinosaur, DeepIso, and 3D-MSNet had similar distributions. For a clearer quantitative evaluation, we calculated the mean quantification bias percentage (|FC ratio – 1| * 100) among samples (Potato set: sample 1 to 7, Human set: sample 5 to 12, Background set: all samples). The calculated results are summarized in the Table A3.8.

Table A3.8: Average FC ratio bias percentage.

	Potato set	Human set	Background set
MaxQuant	**19.2**	22.5	2.5
Dinosaur	25.3	21.8	2.8
DeepIso	28.4	22.6	3.2
PointIso	28.7	30.0	9.1
**3D-MSNet (Ours)**	25.1	**19.4**	**2.2**

3D-MSNet obtained the lowest quantification bias percentage in the human set and the background set. MaxQuant achieved the lowest quantification bias percentage in the potato set. The lower the quantification bias percentage, the higher the quantification accuracy. The accuracy ranking is summarized as follows: 3D-MSNet = MaxQuant > Dinosaur > DeepIso > PointIso.

8. Conclusion

The evaluation rankings of feature detection and quantification evaluation results are shown below:

Feature detection rate: 3D-MSNet > MaxQuant > Dinosaur > DeepIso > PointIso

Feature quantification stability: 3D-MSNet > Dinosaur > MaxQuant > DeepIso > PointIso

Feature quantification accuracy: 3D-MSNet = MaxQuant > Dinosaur > DeepIso > PointIso

Overall, 3D-MSNet achieved the best performance in the evaluation on the Orbitrap XL dataset with the targeted library. 3D-MSNet presented the highest feature detection rate and quantitative stability with high quantitative accuracy.
